# Myosin phosphatase and RhoA-activated kinase modulate arginine methylation by the regulation of protein arginine methyltransferase 5 in hepatocellular carcinoma cells

**DOI:** 10.1038/srep40590

**Published:** 2017-01-11

**Authors:** Adrienn Sipos, Judit Iván, Bálint Bécsi, Zsuzsanna Darula, István Tamás, Dániel Horváth, Katalin F. Medzihradszky, Ferenc Erdődi, Beáta Lontay

**Affiliations:** 1 Department of Medical Chemistry, Faculty of Medicine, University of Debrecen, Debrecen, H-4032, Hungary; 2 MTA-DE Cell Biology and Signaling Research Group, Faculty of Medicine, University of Debrecen, Debrecen, H-4032, Hungary; 3 Laboratory of Proteomics Research, Biological Research Centre, Hungarian Academy of Sciences, Szeged, H-6726, Hungary

## Abstract

Myosin phosphatase (MP) holoenzyme is a protein phosphatase-1 (PP1) type Ser/Thr specific enzyme that consists of a PP1 catalytic (PP1c) and a myosin phosphatase target subunit-1 (MYPT1). MYPT1 is an ubiquitously expressed isoform and it targets PP1c to its substrates. We identified the protein arginine methyltransferase 5 (PRMT5) enzyme of the methylosome complex as a MYPT1-binding protein uncovering the nuclear MYPT1-interactome of hepatocellular carcinoma cells. It is shown that PRMT5 is regulated by phosphorylation at Thr80 by RhoA-associated protein kinase and MP. Silencing of MYPT1 increased the level of the PRMT5-specific symmetric dimethylation on arginine residues of histone 2 A/4, a repressing gene expression mark, and it resulted in a global change in the expression of genes affecting cellular processes like growth, proliferation and cell death, also affecting the expression of the retinoblastoma protein and c-Myc. The phosphorylation of the MP inhibitory MYPT1^T850^ and the regulatory PRMT5^T80^ residues as well as the symmetric dimethylation of H2A/4 were elevated in human hepatocellular carcinoma and in other types of cancers. These changes correlated positively with the grade and state of the tumors. Our results suggest the tumor suppressor role of MP via inhibition of PRMT5 thereby regulating gene expression through histone arginine dimethylation.

Hepatocellular carcinoma (HCC) is one of the most common cancers worldwide and is a leading cause of cancer-related deaths. The molecular mechanism behind the pathogenesis of HCC is poorly understood, although molecular markers and more precise classification would be crucial^1^. One of the potential therapeutic target mechanisms is reversible protein phosphorylation at serine (Ser) and threonine (Thr) residues by the coordinated action of protein kinases and phosphatases. More than 98% of cellular protein phosphorylation occurs at Ser/Thr^2^ and it regulates intracellular signal transduction pathways resulting in profound changes in cellular responses. Many protein kinases are identified as oncogenes and protein dephosphorylation by protein phosphatases may also play a critical role in malignant transformation of cells^3^. Protein phosphatase-1 (PP1) is one representative of the major phospho-Ser/Thr (P-Ser/Thr) specific eukaryotic protein phosphatases. Mammalian genomes contain three different genes that encode five distinct PP1 catalytic subunits (PP1c): PP1c*α1*/*a2*, PP1c*β*/*δ* and PP1c*γ*_1_/*γ*_2_^4^. Several lines of evidence suggest that PP1 activity is required for exit from mitosis, yet may also block cell cycle progression and facilitate apoptosis^5^. Moreover, okadaic acid, a potent and specific inhibitor of PP1 and protein phosphatase 2 A (PP2A) enzymes was described as a powerful tumor promoter that triggers tumor formation in various organs such as liver^6^. Previous studies reported that the nuclear PP1cα and PP1cβ/δ activity significantly decreased in human HepG2 and rat AH13 hepatoma cells compared with primary cultured hepatocytes also suggesting that PP1 is a tumor suppressor in its own right^5^.

It is assumed that free PP1c is not present in the cells, but it performs its diverse biological activities by forming holoenzymes with one of the almost 200 PP1c-binding proteins, termed targeting/inhibitory subunits, in a mutually exclusive manner^7^. The functions of these proteins are to target PP1c to the substrate, to modulate the enzyme activity and to determine the subcellular localization^8^. Myosin phosphatase target subunit 1 (MYPT1) is one of the regulatory proteins that binds to PP1c to form a highly stable holoenzyme referred to as myosin phosphatase (MP). MP is a heterotrimer composed of the β/δ isoform (termed both ways) of PP1c and two non-catalyic subunits, MYPT1 and a 20 kDa subunit with essentially unknown function^9^. It is the dominant form in smooth muscle but is expressed ubiquitously in mammalian tissues^10^. Much of the earlier works have focused on smooth muscle MP, which regulates the phosphorylation level of the 20 kDa regulatory light chains of myosin II, thereby mediating contractile and motile events in these cells^11^. Although numerous cytoskeletal MP substrates have been identified so far^12^ suggesting its role in cell migration and metastasis formation, MYPT1 was also found in the nuclear and microsomal fraction of HepG2 cells^13^, and in the nucleus of neuronal cells^14^. MP plays important roles in nucleus related cellular processes, including mitosis by antagonizing polo-like kinase-1^15^, apoptosis by dephosphorylating and facilitating the nuclear localization of the histone deacetylase protein and repression of *Nur77*, a proapoptotic gene^16^. MP also inhibits oncogenic signaling cascades by modulating the dephosphorylation of merlin tumor suppressor protein, which regulates the Ras-signaling extracellular signal-regulated kinase (ERK) pathway driving tumorigenic transformation^17^. MP also modulates the dephosphorylation of the retinoblastoma protein, which plays a role in G1/S transition of cell cycle^18^. Moreover, MYPT1 KO mice develops a lethal phenotype indicating the crucial role of MP in cell proliferation and gene expression^19^. Thus, the above data suggest that dysregulation of phosphorylation signaling pathways related to MP is directly correlated with a plethora of diseases such as cancer and diabetes.

One of the potential regulatory pathways related to MP function is driven by the RhoA GTPase and its downstream Ser/Thr kinase termed RhoA-activated kinase (ROK) which was shown to phosphorylate MYPT1 at Thr696 and Thr850, thereby causing inhibition of MP activity^20^. Although Thr696 of MYPT1 can be phosphorylated by other kinases, the Thr850 (by human sequence) inhibitory phosphorylation site is dominantly regulated by ROK^21^. Additionally, numerous other downstream targets of ROK have been identified including intermediate filaments, adducin and the dephosphorylation of these substrates at the ROK-specific phosphorylation site are catalyzed by MP^22^. It was shown that initiation of HCC formation^23^ is mediated by ROK and hyperactivation of RhoA/ROK is associated with aggresive tumors with enhanced metastatic features in numerous organs^24^. Moreover, Y27632, the selective inhibitor of ROK suppresses invasiveness of several animal cancers such as HCC^25^. A plethora of studies have suggested that ROK inhibitors might be drug candidates in the treatment of cancer by targetting stromal cells rather than tumor cells^26^.

To elucidate the contribution of the ROK/MP axis in tumorigenesis beyond their already described role in angiogenesis and metastasis our goal was to describe the nuclear function of MP. Our present data indicate a potential tumorsuppressor role for MP in human hepatocarcinoma cells via antagonizing the tumorigenic effect of ROK. Our results shed light on the involvement of ROK/MP enzyme pair in the phosphorylation/dephosphorylation of the protein arginine methyltransferase 5 (PRMT5) via regulating arginine methylation of histones thereby affecting signaling for cancer formation.

## Results

### MYPT1 nuclear interactome: interaction with PRMT5 of the methylosome complex

Initial reports have already described the presence of MYPT1 in the nucleus but its exact subnuclear localization and function remained unknown^13^. MYPT1, PP1cα/γ1 and PP1cδ were distributed in the cytoplasmic and nuclear subcellular fractions (Fig. S1A). Specific activity measured in the presence of 2 μM inhibitor-2 (I2) which is a highly selective inhibitor of PP1, but does not influence PP2A activity^27^ suggested that ~60% of total phosphatase activity of nuclear fraction was due to PP1 (Fig. S1B). Myosin phosphatase was found to be localized to the chromatin and the spliceosomes in the nucleus of HepG2 cells (Fig. S1C). To gain more data for the role of MP, we determined the nuclear interactome of Flag (FT)-MYPT1 by mass spectrometry. Pull-down assays were carried out using FT-MYPT1 protein bound to anti-Flag M2 affinity gel which was incubated with the nuclear extract of HepG2 cells. The FT-MYPT1 binding proteins were eluted from the beads, separated by SDS-PAGE then the protein bands were silver-stained, excised and subjected to LC-MS/MS analysis. Twenty-two potential MYPT1-interacting proteins were identified (Table 1). The peptide sequences identified are presented in Table S1 together with the percentage distribution for cellular roles of their parent proteins (Table S2). Nuclear MYPT1-binding proteins may play roles in RNA processing and splicing and in gene expression (Table 1). Mitogen-activated protein kinase and magnesium-dependent protein phosphatase 1B (PP1B/PP2C) were also among the potential interacting partners of MYPT1. Strikingly, the protein arginine methyltransferase 5 (PRMT5/JBP1), the member of the methylosome complex was also identified. PRMT5 is one of the nine PRMTs in mammalian cells and plays an unique and specific role in the generation of the ω-N^G^, N^G^-symmetric dimethylarginine (SDMA) as a type II PRMT. It has multiple cytosolic and nuclear binding partners and is implicated in the regulation of transcription, RNA transport and cellular signaling^28^.

### PRTM5 of the methylosome complex interacts with MP through the N-terminal region in MYPT1

**Figure 1:**
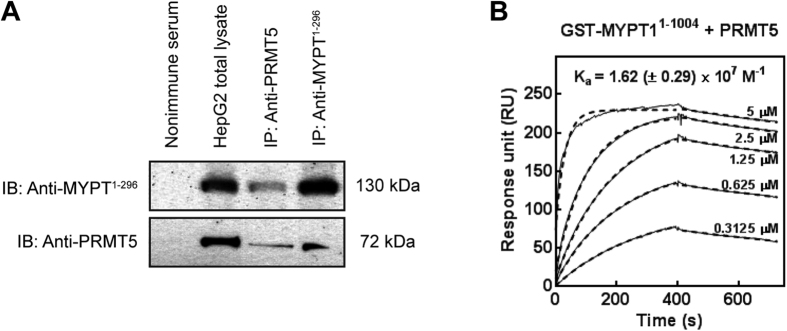
Interaction of MYPT1 with PRMT5. (**A**) Immunoprecipitations were carried out using anti-MYPT1^1-296^ and anti-PRMT5 antibodies as well as nonimmune rabbit serum as negative control. Immunoprecipitates and HepG2 total lysate were analysed by Western blots using antibodies specific for MYPT1 and PRMT5. (**B**) SPR analysis of the interaction of MYPT1 with PRMT5. Full-length GST-MYPT1^1-1004^ was immobilized on anti-GST coupled CM5 sensor chip. FT-PRMT5 was injected over the surfaces in the indicated concentrations. The interaction was monitored using Biacore 3000. The association constant (K_a_) value was indicated in the figure.

Since PRMT5 was identified as a nonspecific interacting protein in affinity purified Flag-tagged complexes from HeLa and tsa cell lysates^29^, we verified the interaction between the nuclear PRMT5 and MYPT1 with additional methods. Anti-MYPT1^1-296^ and anti-PRMT5 antibodies were used to immunoprecipitate target proteins and a possible association between MYPT1 and PRMT5 was assayed in HepG2 cells (Fig. 1A). PRMT5 and MYPT1 were identified in each precipitates as judged by Western blots implying that these two proteins were co-precipitated. No cross reactions were detected in the non-immune serum control (Fig. 1A). Surface plasmon resonance (SPR)-based binding assays were carried out to investigate the molecular background of MYPT1-PRMT5 interaction using full-length GST-MYPT1 as well as a GST-fusion C-terminal fragment of residues 667 to 1004 (GST-MYPT1^667-1004^). These MYPT1 species were bound on SPR sensorchips to immobilized anti-GST and purified FT-PRMT5 was flown through the flow cells as an analyte. PRMT5 formed a stable complex with the full-length GST-MYPT1^1-1004^ in a concentration range of 0.3125–5 μM and the association constant for binding was K_a_ = (1.62 ± 0.29) × 10^7 ^M^−1^ (Fig. 1B), while no signal for binding was obtained with GST-MYPT1^667-1004^ (data not shown). Fitting the sensograms with a 1:1 binding model resulted in overlaps with the experimental curves suggesting a 1. 1 molar ratio in the GST-MYPT1^1-1004^- PRMT5 complex formed. These data demonstrate also that PRMT5 forms a stable interaction with the N-terminal region of MYPT1.

### PRMT5 is a substrate of ROK and MP and its methyltransferase activity is regulated via phosphorylation/dephosphorylation of its Thr80 residue

**Figure 2:**
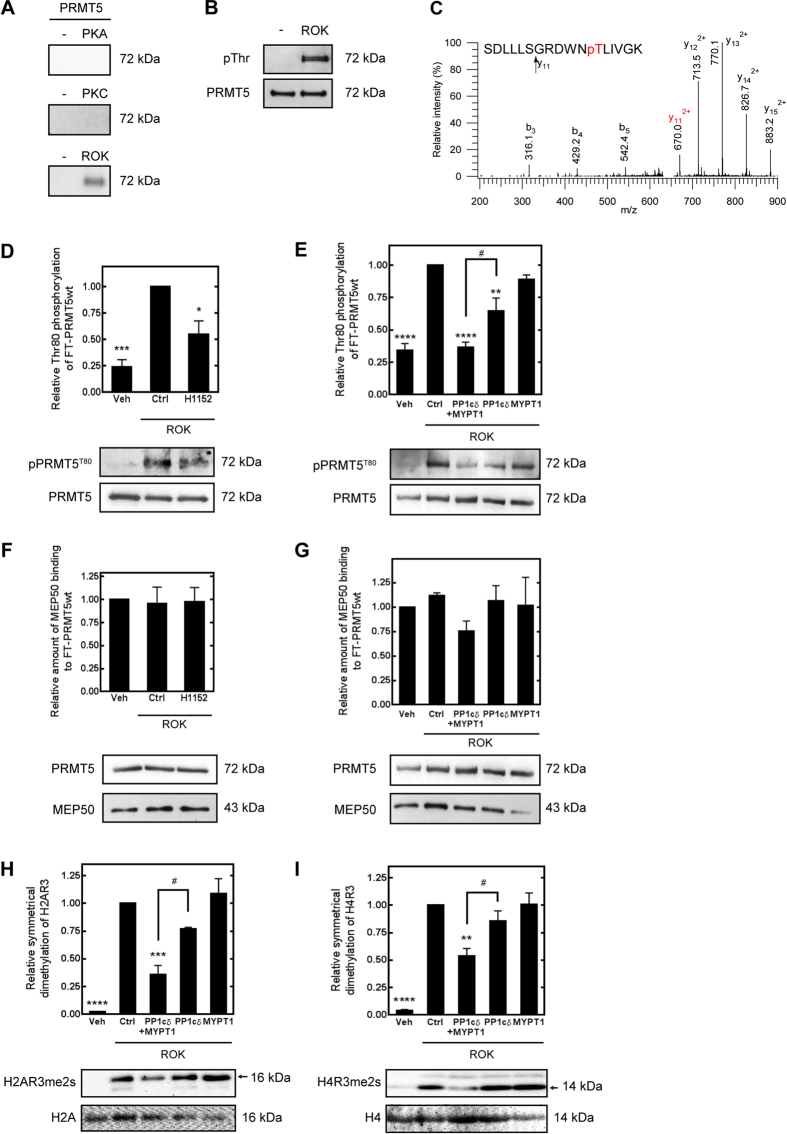
ROK and MP regulate the methyltransferase activity of PRMT5 through phosphorylation/dephosphorylation at Thr80. (**A**) Autoradiograms of PRMT5 phosphorylated in the absence or in the presence of 0.1 μg/ml protein kinase A (PKA, left panel), 0.1 μg/ml protein kinase C (PKC, middle panel) or 0.4 U/ml Rho-associated kinase (ROK, right panel) with ^32^P-ATP. (**B**) Western blot analysis of ROK-phosphorylated PRMT5 using antibody specific for phospho-Thr. After stripping the membrane anti-PRMT5 antibody was applied to detect PRMT5 as an input control. (**C**) Ion trap collision-induced dissociation (CID) spectra of PRMT5 phosphopeptides. CID of m/z: 656.338 (3+) identified as SDLLLSGRDWNpTLIVGK representing [69–85] of the wild type protein. Thr80 was identified as the modification site (see fragment ion y_11_ (phosphorylated)). Peptide fragments are labeled according to the nomenclature by Biemann^56^. (**D**) Effect of ROK inhibitor (10 μM H1152) on the phosphorylation level of PRMT5 during *in vitro* ROK assay. Control samples were prepared in the absence of ROK, positive control samples were prepared in the presence of ROK without ROK inhibitor. Relative phosphorylation level of Thr80 was judged by Western blot using anti- pPRMT5^T80^ antibody and blots for PRMT5 served as loading control. (**E**) Effect of 25 nM FT-MYPT1 and 5 nM rPP1cδ or their combination on the phosphorylation level of PRMT5 at Thr80^80^ as judged by Western blot. Data were compared to ROK-phosphorylated PRMT5. (**F**,**G**) Amount of MEP50 bound to FT-PRMT5 during ROK-phosphorylation (**F**) and dephosphorylation by MP (**G**) compared to unphosphorylated control samples. MEP50 was detected by anti-MEP50 antibody during Western blot and relative amount was normalized to the level of PRMT5. (**H**,**I**) *In vitro* arginine methyltransferase assay of unphosphorylated and ROK-phosphorylated PRMT5 measured by the symmetric dimethylation level of histone H2A Arg3 (H2AR3me2s, F) or histone H4 Arg3 (H4R3me2s, G) in the presence of 25 nM FT-MYPT1, 5 nM rPP1cδ or their combinations. Gels have been processed under the same experimental conditions. Values represents mean ± SEM; **p < 0.01, ***p < 0.001, ****p < 0.0001, ^#^p < 0.05, one-way ANOVA followed by Tukey’s multiple comparison test, n = 3.

The putative phosphorylation sites of PRMT5 were screened and the identities of protein kinases that may target these sites were predicted by a P-Si Predictor alogrithm developed by Kinexus (http://www.phosphonet.ca/). Protein kinase A (PKA), protein kinase C (PKC) and ROK were suggested as potential phosphorylating enzymes therefore they were probed in *in vitro* phosphorylation assays. The autoradiogram in Fig. 2A shows that PRMT5 was phosphorylated by ROK but not by PKA or PKC in kinase assays when radioactive ATP (γ- ^32^P-ATP) was used as phosphoryl donor substrate. Western blot analysis of ROK-phosphorylated PRMT5 by antibody specific for phosphorylated Thr (Fig. 2B) indicated that ROK phosphorylates PRMT5 definitely on Thr residue. Thr80 residue was identified as a ROK phosphorylation site in PRMT5 by mass spectometry analysis of ROK-phosphorylated FT-PRMT5 samples compared to non-phosphorylated ones (Fig. 2C). Ser15/16, Thr67 were Ser69 were also identified as potential phosphorylation sites of PRMT5 from LC-MS/MS data. However, only Thr80 phosphorylation was unambiguously linked to the ROK-treatment since the phosphorylation of Ser15/16 was also identified in control samples which were incubated without ROK and the Thr67 and Ser69 phosphorylation sites were infirm even after the enrichment using titanium-oxide chromatography (Fig. S6.).

ROK-specific phosphorylation of PRMT5^T80^ was confirmed by ROK-assay (Fig. 2D, Fig. S3A) in which the relative Thr80 phosphorylation level of wild type PRMT5 determined by anti-phospho-PRMT5^T80^ antibody (anti-pPRMT5^T80^) was significantly decreased in the presence of H1152, a selective Rho-kinase inhibitor. Alanine mutant of PRMT5^T80^ (PRMT5^T80A^) was generated by site-directed mutagenesis and phosphorylation of this mutant was probed in ROK assay in the presence and absence of H1150. As judged with anti- anti-PPRMT5^T80^ antibody on Western blot no signal was detected confirming the Thr80 specificity of ROK in PRMT5 phosphorylation (Fig. S3B).

To prove the regulatory role of MP on PRMT5, FT-PRMT5 was phosphorylated by ROK and *in vitro* phosphatase assays were carried out using recombinant PP1cδ and purified FT-MYPT1 proteins or their combination representing MP holoenzyme (Fig. 2E). Western blot data using anti-pPRMT5^T80^ showed that the FT-MYPT1 had no effect on the phosphorylation level of PRMT5 at Thr80, whereas recombinant PP1cδ or the mixture of PP1cδ and FT-MYPT1 caused ~14% and ~40% decrease in phospho-PRMT5^T80^, respectively comparing to the ROK-phosphorylated samples. The increased dephosphorylation of PRMT5 at Thr80 by PP1cδ in presence of FT-MYPT1 indicates that the phosphorylated PRMT5 is a substrate of MP holoenzyme and MYPT1 has a targeting role in this dephosphorylation process.

**Figure 3:**
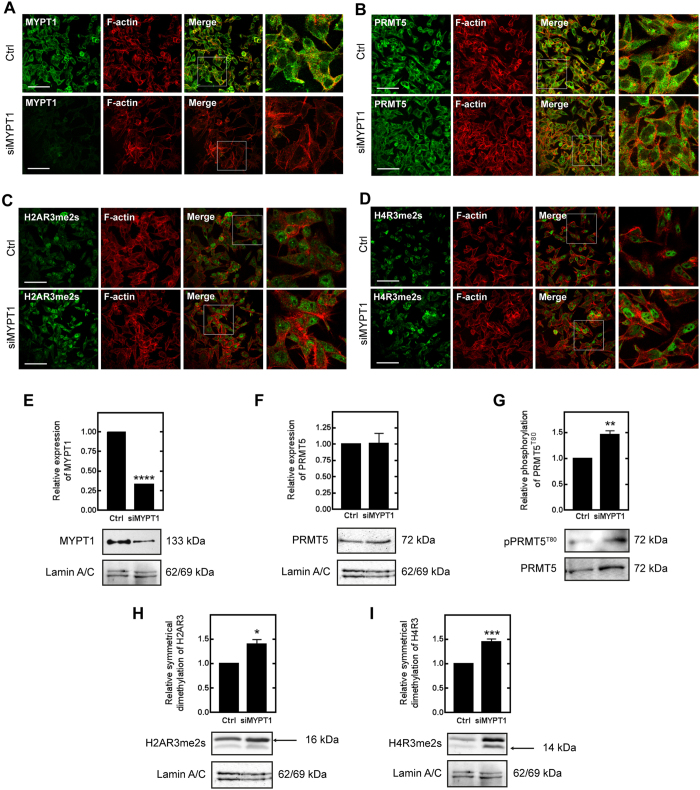
Effect of MYPT1 silencing on the methyltransferase activity of PRMT5 in HepG2 cells. Immunofluorescent staining of non-target control (Ctrl) and MYPT1-silenced (siMYPT1) cells using antibodies specific for MYPT1^1-296^ (**A**), PRMT5 (**B**), histone H2A symmetric dimethyl Arg3 (H2AR3me2s (**C**) and histone H4 symmetric dimethyl Arg3 (H4R3me2s (**D**) and actin as inducated in the figures. Scale bars: 50 μM. Enlargement of framed regions in merged images are shown on the right on A, B, C and D panels. Nuclear fractions of non-target control (Ctrl) and MYPT1-silenced (siMYPT1) HepG2 cells were prepared and analysed by Western blot using anti-MYPT1^1-296^ (**E**), anti-PRMT5 (**F**), anti-pPRMT5^T80^ (**G**), anti-histone H2A symmetric dimethyl Arg3 (**H**) and anti-histone H4 symmetric dimethyl Arg3 (**I**) specific antibodies. The relative expression of MYPT1 or PRMT5 and the relative symmetric dimethylation level of H2AR3 or H4R3 were normalized to lamin A/C as internal control. Samples derived from the same experiment and the blots were processed in parallel or assayed after stripping. The relative phosphorylation level of PRMT5^T80^ was normalized to the expression level of PRMT5 and then to lamin A/C as internal control by densitometry. Mean ± SEM; n = 3; *p < 0.05, **p < 0.01, ***p < 0.001, ****p < 0.0001 by student *t-*test.

To determine the effect of the phosphorylation of PRMT5 at Thr80 on its methyltransferase activity we assayed PRMT5 activity by determining symmetric dimethylation of histone 2A and 4 (H2A and H4) on their common arginine 3, the so called “R3 motif”^30^. No significant changes in symmetrical dimethylation of H2AR3 or H4R3 were detected when FT-MYPT1 without PP1cδ was used (Fig. 2H and I). Nevertheless, in accordance with the rate of PRMT5-dephosphorylation represented in Fig. 3G symmetrical dimethylation of H4R3 was decreased by 15% and 46% when PP1cδ and the mixture of FT-MYPT1 and PP1cδ was applied during methyltransferase assay. Changes in the symmetrical dimethylation level of H2AR3 (Fig. 2F) were even more profound, 23% adding PP1cδ and 67% adding the mixture of PP1cδ and FT-MYPT1. Since MEP50 is an essential component of the methyltransferase activity of PRMT5, we tested if the phosphorylation at Thr80 increases the affinity of MEP50 to PRMT5 resulting the enhanced activity upon ROK phosphorylation. The phosphorylation by ROK with or without the inhibition of ROK by H1152 had no effect on the amount of the MEP50 bound to the wt PRMT5 (Fig. 2F.). The relative amount of MEP50 binding to wt PRMT5 showed no significant differences either upon ROK phosphorylation or the dephosphorylation by MP (Fig. 2G.) suggesting that the phosphorylation of PRMT5 at Thr80 has no effect on MEP50 binding. These data indicate that Thr80 residue is a regulatory phosphorylation site of PRMT5 and the phosphorylation increases, while the dephosphorylation decreases its methytransferase activity.

### Silencing of MYPT1 increased H2A and H4 Arg3 dimethylation and PRMT5 phosphorylation at Thr80 in HepG2 cells

Histone 2AR3 and −4R3 are symmetrically dimethylated by PRMT5 which shifts the balance from an activating asymmetric (ADMA) to a suppressive symmetric dimethylarginine (SDMA) mark at these motifs with respect to gene expression^31^. The fact that MP dephosphorylated PRMT5 at the ROK-specific phosphorylation site *in vitro* led us to examine whether MP plays a role in the regulation of gene expression through the arginine methylation of H2A and 4 at the gene repression sites *in vivo*. We first transfected HepG2 cells with MYPT1-specific siRNAs (siMYPT1) or non-targeting siRNA as a control. SiMYPT1 was able to lower the protein expression of MYPT1 by >70% (Fig. 3E) without causing any significant decrease either in the survival (Fig. S4A) or the caspase-3 activity (Fig. S4B) as signs for apotosis of HepG2 cells. Upon MYPT1-silencing drastic changes in cell morphology and in formation of stress fibers were detected by immunofluorescent staining (Fig. 3A). PRMT5 was localized to the cytosolic as well as to the nuclear fraction (Fig. 3B). MYPT1-silencing had no effect on either the protein expression (Fig. 3F) or on the subcellular localization of PRMT5, however, the phosphorylation of PRMT5 at Thr80 was increased by 46% (Fig. 3G). Moreover, we examined the effect of siMYPT1 on the level of dimethylation of H2AR3 (Fig. 3H) and H4R3 (Fig. 3I) and it was elevated by 40% and 45%, respectively, whereas the expression of both histone proteins remained constant. The fluorescence intensity of the dimethylation of H2AR3 (Fig. 3C) and H4R3 (Fig. 3D) increased followed by MYPT1-silencing. Fluorescent mean intensity of H2AR3 symmetrical dimethylation increased from 12.8 to 21.79 (arbitrary unit) due to MYPT1-silencing. Difference in symmetrical dimethylation was more considerable in case of H4R3 (from 8.19 to 22.58) compared to non-significant intensity changes of H2A or H4 expression (Table S6). To verify our data we applied the nuclear extract of non-target and MYPT1-silenced HepG2 and MCF7 cells on quantitative methyltransferase assay (Fig. S5A and B). Knocking down MYPT1 increased the specific activity of PRMT5 by ~65% comparing to the control samples. Taken together, our results reflect the physiological regulatory role of MP in the methyltransferase activity of PRMT5 and thus influence the dimethylation of histone respression marks.

### Silencing of MYPT1 results in altered gene expression pattern and downregulates the expression of tumor suppressors in HepG2 cells

**Figure 4:**
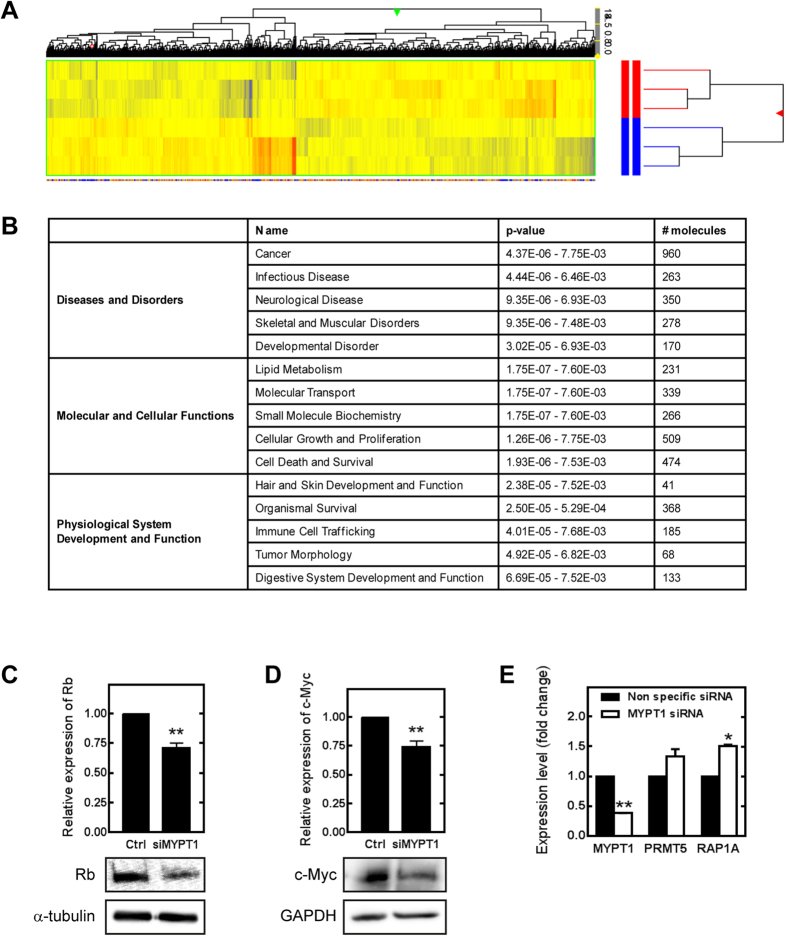
Microarray analysis of MYPT1-silenced HepG2 cells. (**A**) Heat map of genes related to MYPT1 silencing in HepG2 cells by microarray analysis. The color code for the signal strength is shown in the box at the bottom in which induced genes are indicated by red and repressed genes are indicated by blue. (**B**) The onthology of the related genes and their classification by GO terms. Detection of retinoblastoma protein (**C**) and c-Myc (**D**) protein expression changes due to MYPT1 silencing from HepG2 whole cell lysates by Western blot. Protein levels were quantified by densitometry normalized to α-tubulin or GAPDH expression level. Samples derived from the same experiment and processed in parallel. RT-PCR analysis of MYPT1, PRMT5 and RAP1A mRNA levels in non-specific siRNA treated and MYPT1 silenced HepG2 cells (**E**). GAPDH was used as an invariant gene. Values are mean ± SEM from three independent experiments; *p < 0.05, **p < 0.01 by student *t-*test.

To more precisely define the role of MP in functions related to the regulation of dimethylation of histone proteins, we performed microarray studies using the Affymetrix Human Gene 1.0 ST Array for the analysis of gene expression. Over 39,000 transcripts/single array were examined encompassing the entire expressed human genome. Total RNA was isolated from control and MYPT1-silenced (siMYPT1) HepG2 cells (Fig. 4). The comparison of control and siMYPT1 samples identified 2728 genes differentially regulated between the two groups. Complete lists of genes are available at www.figshare.com website. Table S7 shows a list of genes that were up- or downregulated by more than 1.5-fold (*n* = 3) in the siMYPT1 samples when compared to gene expression pattern. Hierarchical cluster analysis was performed on all differentially expressed genes using average linkage with Pearson’s dissimilarity and the number of induced and repressed genes were presented in a heat map (Fig. 4A). Bioinformatic analysis was assigned to different canonical pathways such as LPS/IL-1 mediated inhibition of RXR function (PPAR α, γ, IL1R, JUN), antioxidant action of vitamin C (STAT5, MAPK3 and 9), cell cycle regulation (Not7, PPP2C, CDK2, E2F3, Rb protein) as well as IL-4 and IL-8 signaling. Knocking down of MYPT1 also influenced molecular and cellular functions such as lipid metabolism, molecular transport, cell growth and differentiation and cell death. Additionally, the siMYPT1-related genes were analyzed for identifying diseases and disorders relevant to the list. Strikingly, 36% of all related genes (960) were linked to cancer disease such as lymphohematopoietic, liver and breast cancer and renal-cell carcinoma formation. Genes related to infectious diseases and developmental disorders were also identified in 10% and 6%, respectively. Taken together, microarray analysis identified a number of signaling pathways strongly point to a role of MP in the regulation of gene expression.

The evaluation of genes related to MYPT1-silencing revealed the significant downregulation of several tumor suppressors and transcription factors. One of them is the retinoblastoma protein (pRb) that plays a pivotal role in the negative control of the cell cycle and in tumor progression as a major regulator of G1 checkpoint^32^. The other is c-Myc that regulates the cell cycle progression and cellular transformation as a multifunctional transcription factor^33^. The protein expressions of pRb and c-Myc were significantly decreased upon MYPT1 silencing and these were also confirmed by Western blot analysis (Fig. 4C and D). To clarify fully the connection between the MP activity and the PRMT5-regulated gene expression we screened for genes related to both PRMT5 and MP in the database of the siPRMT5^34^ and siMYPT1 microarray analysis, respectively. Rap1 gene expression was 2.3 fold reduced upon PRMT5 silencing in human embryonic stem cells and the expression of this gene was found to be significantly increased in siMYPT1 cells (Fig. 4E).

### Myosin phosphatase is involved in the regulation of gene expression through the suppression mark of PRMT5 on histone 2A and 4 in human cancer

**Figure 5:**
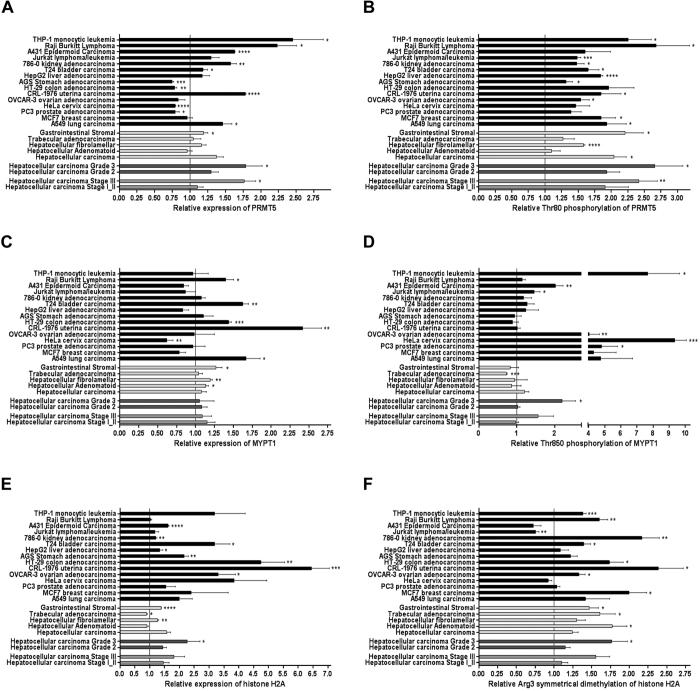
Protein expression profile of HCC and other cancer cell lines. Reverse phase protein microarray analysis was conducted to study the changes in protein expression and posttranslational modification of PRMT5 (**A**,**B**), MYPT1 (**C**,**D**) and histone H2A (**E**,**F**) proteins in normal and tumor human cell lysates. Human HCC samples (n = 20) were grouped based on their clinically verified stage (middle grey columns) or grade (dark grey columns) classification of tumor. Average of HCC samples irrespectively of grouping and other types of metastatic liver cancer tissues are shown next to each other (light grey columns). Protein microarray contains 15 cancer cell lines and normal tissue lysate of controls of corresponding organs in triplicates (black columns). Value of 1 means average of relative expression, phosphorylation or symmetrical dimethylation of the given protein or residue to the corresponding non-tumor samples. Blots were processed under the same experimental procedure. Datas are mean ± SEM; *p < 0.05, **p < 0.01, ***p < 0.001, ****p < 0.0001 by student *t-*test.

The microarray analysis indicated that in case of decreased level of MP cancer-related processes dominate. Reverse phase protein microarray analysis was conducted in multiple tissue lysates of patients for the validation of changes in the protein levels and their posttranslational modifications in cancer. Tumor tissue lysates obtained from a large group of human patients with grade 2 and 3 and state II and III (Fig. 5 dark grey columns) hepatocellular carcinoma (n = 20), four different types of metastatic liver cancers (Fig. 5 light grey columns) as well as 15 other types of cancer cell lines with their adequate normal cell type controls (Fig. 5 black columns) were analysed using high throughput screening for multiple protein targets such as PRMT5 (Fig. 5A), pPRMT5^T80^ (Fig. 5B), MYPT1 (Fig. 5C), pMYPT1^T850^ (Fig. 5D), histone 2A (Fig. 5E) and histone 2A symmetric arginine methylation (Fig. 5F).

The protein expression of PRMT5 showed an increase in ~75% of the investigated cancer tissues comparing to the healthy controls. THP-1 monocytic leukemia and Raji Burkitt lyphomoma cells presented the largest increase (Fig. 5A). The relative phosphorylation of PRMT5 at Thr80 in cancer tissues were significantly elevated in all cases escpecially in the leukemia, lung, colon and breast cancer carcinoma as well as in HCC tissues (Fig. 5B). The relative MYPT1 expression exhibited a diverse expression pattern since it was significantly downregulated in few cases (HeLa cervix and A549 lung carcinoma) but in uterine carcinoma it showed a twofold increase. However, MYPT1 expression did not change in the majority of cancer types (65%). Since MP holoenzyme activity is regulated through the phosphorylation of MYPT1 at the Thr850 inhibitory site, we were able to draw conclusions about the activity of PP1M in tumor cells based on the phosphorylation state of MP. Increased phosphorylation level of MYPT1^Thr850^ was found in almost all cases up to a 10–12-fold elevation in breast and lung carcinomas (Fig. 5D). The overexpression of histone proteins in cancer cells and the elevated symmetric dimethylation of them have been already described in colorectal, glioblastoma and numerous other cancer types^35^,^36^. We also verified either no changes (Burkitt lymphoma, hepatocellular adenomatoid and trabecular adenocarcinoma) or significant elevation in histone protein expression (Fig. 5E) and 85% of all examined cancer types showed an increase in the repressive Arg3 symmetrical dimethylation mark. Our results imply that the decreased activity of MP bears an obvious relation to the increased activity of PRMT5 and the increased gene repression mark on histones in almost every investigated cancer types particularly in bladder and colon carcinoma, MCF7 breast cancer, THP1 monocytic leukemia, kidney adenocarcinoma and in hepatocellular carcinoma. Except for the expression of MYPT1 in all other cases, the protein expression as well as the phosphorylation of PRMT5 at Thr80, and the arginine methylation of histone 2A and 4 increased in the higher grade and stage in hepatocellular carcinoma tissues (n = 11–13) exhibiting a strong correlation to the grade and state of the tumor. These data suggest the involvement of the ROK/MP/PRMT5/histone dimethylation pathway in tumorigenesis.

## Discussion

In the present study we have characterized a novel mechanism of tumorigenesis governed by ROK and MP in hepatocarcinoma cells (HCC). We have identified Thr80 in PRMT5 as the target of ROK/MP and the phosphorylation/dephosphorylation of this site is shown to modulate the activity of PRMT5. At high phosphorylation of PRMT5 at Thr80 symmetric dimethylation of the histone R3-motif is preferred which accompanied by tumorogenesis, while dephosphorylation by MP has an opposite effect. Therefore, MP could be assumed as a tumorsuppressor in HCC. The interplay between MP and PRMT5 is also supported by interaction analysis. First, the presence of PRMT5 in the MYPT1 nuclear interactome is appealing for an interacting protein complex. Second, SPR-based *in vitro* binding assay identified a stable interaction between MYPT1 (the large targeting subunit of MP) and PRMT5 and it was verified that PRMT5 binds to the N-terminal region of MYPT1.

Our findings show that ROK/MP counteract on the Thr80 phosphorylation site of PRMT5 regulating its methyltransferase activity both *in vitro* (Fig. 2) and *in vivo* (Fig. 3). The PRMT5 protein includes multiple tyrosyl and seryl/threonyl residues that could be potentially targeted by phosphorylation. PRMT5 can be phosphorylated by a constitutively active janus kinase 2 mutant at the tyrosyl 304 and 307 residue resulting in the disruption of the interaction between MEP50 and PRMT5 and the inhibition of the activity of PRMT5 in patients with myoproliferative neuroplasm^37^. The ROK/MP regulated Thr80 residue and its surrounding peptide sequences are not part of the methyltransferase domain of PRMT5 and play no role in the formation of the heterooctameric structure of PRMT5_4_:MEP50_4_ based on the crystal structure of human PRMT5^38^. PRMT5^T80^ is located in a peptide sequence (GRDWNTLI/VV) well conserved among vertebrates but not in invertebrate orthologs. Furthermore, PRMT5 exhibits no sequence homology with PRMT1-4 enzymes^39^ suggesting a selective regulatory function of its phosphorylation at Thr80. The fact that PRMT5-Thr80 phosphorylation increases the activity of the enzyme without the recruitment of more MEP50 into the complex supports the hypothesis that this modification triggers changes either in the substrate- or in the S-adenosylmethionine methyl donor binding sites. Our findings provide a yet unrecognized signaling mechanism which regulates PRMT5 activity directly with posttranslational modification.

Histone tail modifications are major components of the epigenetic regulation of gene transcription. It is well established that PRMT5 has preference for the symmetrical dimethylation of H4R3, H2AR3 and H3R8 shifting the balance to a suppressive mark^40^,^31^. The symmetrically dimethylated R3-motif recruites the DNA methyltransferase DNMT3A to chromatin domains via its ADD domain to suppress gene expression thus representing epigenetic silencing of gene expression^41^. We provided several lines of evidence that silencing of MYPT1 results in a global change in gene expression through the activation of PRMT5 and by the indirect modulation of the “R3-motifs” of H2A and H4. Moreover, the general assumption that PRMT5 functions as a transcriptional corepressor is not definite since symmetric-dimethyl-arginine-containing protein(s) can specifically associate with the IL-2 promoter after T-cell activation and increase gene expression^42^. Our observation that cancer-related pathways are upregulated in HCC upon MYPT1 silencing is the results of the higher expression and hyperactivation of PRMT5, which suppresses gene and protein expression of several tumor suppressors or increasing that of potential oncogenes. First, the tumor suppressor pRb expression was significantly decreased in siMYPT1 HepG2 cells suggesting the anti-tumor role of MYPT1 in HCC. This is well in line with the findings that PRMT5 overexpression triggered the suppression of the transcription of the pRb in leukemia and in lymphoma cell^43^. Second, c-Myc as a global transcriptional regulator can activate genes involved in cell proliferation and growth but it is also able to repress genes involved in cell cycle and adhesion^44^ raising the possibility of a yet-unexplored mechanism used by tumors to suppress differentiation and potentiate agressiveness. C-Myc expression is significantly lower in HCC than in nontumor tissues and is inversely proportional to the grade of differentiation^45^. We found that MYPT1 silencing resulted in a decrease in c-Myc gene and protein expression suggesting again the tumorsuppressor effect of this MP subunit, and presumably MP itself, in HCC. Finally, we also found that Rap1, one of the target genes induced by PRMT5^34^ was upregulated in siMYPT1 HepG2 cells (Fig. 5E). Rap1 functions as a genomwide transcriptional regulator and a critical player in tumor malignancy. The fact that inhibition of Rap1 interfered with tumor development and induced higher apoptosis rates^46^ also suggest that silencing of MYPT1 mimicks the protein expression pattern of cancer cells and the tumor suppressor activity of MYPT1 and therefore MP.

Previous studies speculated that constant and unbalanced phosphorylation of MYPT1 at Thr850 might be related to cancer formation^47^. Based on the phosphorylation of MYPT1^T850^ we confirmed that the activity of the nuclear MP decreased, the activity of PRMT5 phophorylated at Thr80 increased and PRMT5-specific repressive R3-dimethylation increased in HCC and in other cancer cell lines suggesting the fundamental role of this pathway in tumorigenesis. Since both the stage and the grade of the tumors were directly related to the activity of this pathway, we hypothesized that this is an action governed by ROK. The downregulation of RhoA, an upstream activator of ROK^48^ and the direct inhibition of ROK by Incarvine C and Y27632 decreased the phosphorylation of MYPT1 at Thr850 in lung cancer and HCC^49^, respectively, and the ROK-phosphorylated MYPT1 was localized not only in the cytoplasm but in the nucleus as well^50^.

**Figure 6:**
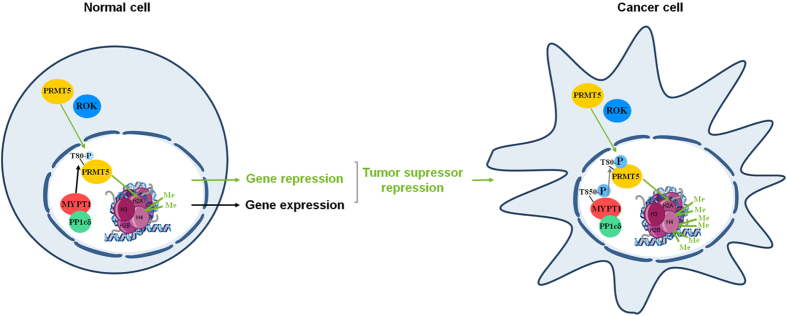
MP plays a tumorsuppressor role and antagonizes ROK in HCC. We hypothesize that MP and ROK are involved in gene expression. Under normal conditions MP modulates symmetrical dimethylation of histone core proteins in cell nucleus via dephosphorylation of PRMT5 at its activatory phosphorylation site (Thr80) causing changes in gene expression. In tumor cells inhibitory phosphorylation of MP on Thr850 is increased leading to higher phosphorylation level of PRMT5 at Thr80 by ROK. Activated PRMT5 provokes gene repression by raising symmetrical dimethylation of histone H4 Arg3 that triggers proto-oncogene activation and tumor formation.

PRMT5 was overexpressed in multiple tumor types including leukemia, lymphoma, lung^27^, colorectal and breast cancer as well as in HCC and it resulted in increased symmetrical dimethylation on H2A and H4 R3-motifs^35^. The prognosis of patients with HCC is associated with the expression level of PRMT5. The overexpression of PRMT5 increased the symmetric dimethylation of histone R3 motifs and we provided evidence that it is related to the enhanced phosphorylation level of PRMT5 at Thr80 (Fig. 6).

In this study we have provided new insights into the regulation of tumor suppressor pathways demonstrating that MP is a negative regulator of the PRMT5 oncoprotein. MP possesses a dual function in cancer-related pathways as it is not only a cytoskeletal regulator governing cell migration and metastasis but it also functions as a key regulator of gene expression and has anti-tumor features. Taken together, the “MP/PRMT5/histone R3” pathway plays a critical role in tumorigenesis and cancer development, therefore the pharmacological interventions of this pathway by targeting MP may have important and promising clinical implications in therapeutics.

## Material and Methods

### Chemicals and antibodies

All chemicals were obtained from Sigma Aldrich unless otherwise indicated. Antibodies used are listed in the Supplementary Information Materials and Methods section. Buffer compositions are described in Supplementary Materials.

### Recombinant proteins

PP1cδ, GST-MYPT1^1-1004^ and GST-MYPT1^667-1004^ were expressed in *E. coli* and purified as described before^51^,^52^. FT-PRMT5 (Sino Biological) for SPR and phosphorylation assays for MS analysis was free from MEP50 (Fig. S2C). FT-PRMT5wt (FT-PRMT5wt), -PRMT5^T80A^ and FT-MYPT1 was purified from transfected tsA201 lysates using anti-Flag M2 affinity gel (Sigma Aldrich) applying the manufacturer’s protocol. FT-PRMT5wt and -PRMT5^T80A^ proteins were bound to anti-Flag M2 affinity gel during kinase and phosphatase assays and *in vitro* protein arginine methyltransferase assays. FT-PRMT5 was in complex with MEP50 (Fig. S2C). PP1c-free FT-MYPT1 was produced for the execution of phosphatase assays and *in vitro* protein arginine methyltransferase assays. The FT-MYPT1 bound to the anti-Flag M2 affinity gel was incubated with MYPT1^1-296^ peptide^13^ several times to dissociate the PP1c subunit then FT-MYPT1 was eluted from the beads with 300 μg/ml Flag-peptide (Sigma Aldrich). The lack of PP1c in the FT-MYPT1 preparation was validated by Western blot with anti-PP1c antibody and phosphatase assay with 32P-MLC20 substrate, The purity of all the PP1cδ (Fig. S2D), GST-MYPT1^1-1004^ and GST-MYPT1^667-1004^ (Fig. S2A) were jugjed by sodium dodecyl sulfate-polyacrylamide gel electrophoresis (SDS-PASGE).

### Cell cultures, transient transfections and silencing of MYPT1

HepG2 cells and human embryonal kidney (tsA201) cells from The European Collection of Cell Cultures were grown as described^13^. All materials used for cell culture were purchased from PAA Laboratories, AustriaThe endogenous MYPT1 protein level was knocked down using double-stranded siRNAs (Table S5, Thermo Scientific Inc.) and a non-target sequence (ON-TARGETplus Non-targeting siRNA #1 from Thermo Scientific Inc.) with the G–C content of siMYPT1 sequences was used as a control. TsA201 cells were transfected with pReceiver-M11 plasmids for overexpression. Protocols are detailed in the Supplementary Methods. The cell lysis and protein measurement were performed as described before^14^.

### HepG2 fractionation method

HepG2 cells were grown to 80% confluency, washed with ice-cold PBS, collected and centrifuged at 4 °C at 1,500 × g for 3 min. After the removal of PBS cells were homogenized in 400 μl buffer A by suspending with a 26 gauge needle 10 times using a 1 ml syringe. The lysate was vortexed for 15 sec. The efficiency of lysis was judged by trypan blue staining monitoring intact nuclei. The lysate was centrifuged at 4 °C at 16,000 × g for 1 min and the supernatant was used as cytosolic fraction. The pellet was resuspended in 200 μl buffer A and passed through the 26G needle 10 times as a washing step. After centrifugation at 4 °C at 16,000 × g for 1 min 100 μl buffer B was added to the pellet and sonicated and used as nuclear fraction.

### Cell viability and caspase assays

HepG2 cells were grown in 96-well plates to determine viability by MTT assay and caspase-3 activity and were carried out as detailed before^53^.

### Immunoblotting

SDS-PAGE and immunoblotting were carried out as describe previously^13^. For quantitative comparisons samples derived from the same experiments. For quantification of PRMT5-Thr80 phosphorylation membranes were stripped and assayed for PRMT5 content as it is described on the Supplementary Methods. Internal controls were either assayed on the same gel or were processed in parallel. Densitometry of the proteins of interest were performed by Image J. 1.46 and normalized to an internal control protein and plotted as relative numbers as detailed in figure legends.

### Immunofluorescence microscopy

HepG2 cells were grown on rat tail collagen-coated coverslips (BD Biosciences) and fixed using 4% paraformaldehyde and treated as described^53^. Primary and secondary antibodies were applied in 1:100 and 1:200 dilutions, respectively. DAPI (1:2000) were added as a nuclear marker as indicated in figure legends. Images were taken by Leica X8 confocal microscope and were processed using Leica X8 software program and PhotoShop Imaging software.

### Surface Plasmon resonance (SPR)

SPR-based binding studies were carried out using a Biacore 3000 instrument to monitor the interaction of MYPT1 with PRMT5. GST-MYPT1^1-1004^ and GST-MYPT1^667-1004^ were immobilized on anti-GST coupled CM5 sensorchip^54^. FT-PRMT5 was injected over the surfaces in various concentrations and the responses recorded. Binding of FT-PRMT5 was characterized by kinetic parameters and association constant (K_a_) values determined by BIAevaluation 3.1 software.

### Site-directed mutagenesis

Expression vector pReceiver-M11 encoding wild-type human full-length PRMT5 (NM_006109.3) transcript variant 1 was purchased from GeneCopoeia Inc., USA. Point mutations of PRMT5^T80A^ in pReceiver-M11 were performed using QuickChange II XL Site-Directed Mutagenesis Kit according to the manufacturer’s instructions (Agilent Technologies). Primer pairs are listed in Table S3. The sequence of mutants were verified by DNA sequencing, primers are listed in Table S4.

### Immunoprecipitation and pull-down assays

For LC-MS/MS analysis of FT-MYPT1 interacting proteins FT-MYPT1 and Flag-peptide as control were bound to anti-Flag M2 affinity gel. The samples were precleared on the anti-Flag M2 affinity gel prior to affinity isolation. After washing with Tris-buffered saline solution (TBS) three times, beads were incubated with HepG2 nuclear extract for 2 hr on 4 °C. The FT-MYPT1 was eluted with 300 μg/ml Flag-peptide from the beads with the anchored proteins. Eluted samples were subjected to SDS-PAGE. Bands of interest were visualized by MS compatible silver staining and the proteins were subjected to in-gel digestion using trypsin. No proteins other than human keratin contamination were identified from the control samples.

For immunoprecipitation (IP) anti-MYPT^1-296^ and -PRMT5 antibodies were incubated with Protein-A Sepharose (GE Healthcare) as described before^14^. The blots were developed with anti-MYPT^1-296^and anti-PRMT5 antibodies followed by Clean-blot IP detection reagent (HRP).

### *In vitro* protein arginine methyltransferase assay

FT-PRMT5wt was bound to anti-Flag M2 affinity gel following phosphorylation by protein kinases and was applied for the methylation assay. 2 μM S-adenosyl-L-methionine and 0.02 mg/ml of histone mixture from calf thymus (Roche) were added to the reaction in a total volume of 50 μl. Methylation reaction was allowed to proceed for 2 hours at 30 °C in the presence or in the absence of 5 nM recombinant PP1cδ^51^, 25 nM FT-MYPT1 or the combination of PP1cδ and FT-MYPT1 (assumed as MP holoenzyme). Products of the methylation reactions were analysed by Western blots using antibodies specific for symmetrical dimethylation of H2A Arg3 and H4 Arg3. Membranes were stripped on 50 °C for 30 min in stripping buffer in every case and were assayed with anti-H2A and anti-H4 antibodies as input controls. Changes of symmetrical dimethylation levels were calculated by densitometry normalized to histone H2A and histone H4 internal controls. Methyltransferase assay was also carried out with colorimetric Epigenase PRMT methyltransferase type II-specific assay kit (Epigentech) following the instructions of the manufacturer.

### Protein kinase and protein phosphatase assays

Phosphorylation of recombinant human PRMT5 (Sino Biological Inc.) was initiated by 0.2 mM ^32^P-ATP or for LC-MS/MS analysis by 0.5 mM ATP in the absence (control) or in the presence of 0.4 U/ml ROK (Upstate, Millipore), 0.1 μg/ml PKA or 0.1 μg/ml PKC at 30 °C for 120 min. ROK was applied in buffer C and all kinase assay buffers contained 1 μM mycrocystin-LR (MC-LR). PKA and PKC assay buffer are described in Supplementary Methods. The reaction mixtures were subjected to SDS-PAGE and bands were detected by autoradiography or visualized by MS/MS compatible silver staining and were cut out and subjected to LC-MS/MS analysis.

Phosphorylation reaction by ROK (0.4 U/ml) was also carried out in the absence or in the presence of 10 μM H1152 with 0.5 mM ATP, 5mM MgCl_2_ and 1 μM MC-LR at 30 °C using FT-PRMT5wt bound to anti-Flag M2 affinity gel. After 15 min incubation kinase assay medium was removed and beads were washed by TBS. Dephosphorylation of ROK-phosphorylated FT-PRMT5wt bound to the beads (the product of the kinase assay) was initiated by adding 25 nM FT-MYPT1, 5 nM rPP1cδ or the combination of these two proteins at 30 °C for 15 min, then the beads were washed by TBS and the bound (eluted??) proteins were subjected to SDS-PAGE. Bands were detected by autoradiography or visualized by MS/MS compatible silver staining. Bands of PRMT5 were cut out and subjected to in-gel digestion using trypsin. Approximately 80% of the peptide mixtures were subjected to phosphopeptide enrichment using TiO_2_^55^, then the phosphopeptide fractions as well as the remaining 20% of the original samples were analyzed by data-dependent LC-MS/MS using an Orbitrap Elite mass spectrometer (MS spectra acquired in the Orbitrap, MS/MS spectra in the linear ion trap). Phosphorylation level of Thr80 of PRMT5 was detected by phospho-PRMT5^T80^ specific antibody on Western blots and analysed by densitometry normalized to the PRMT5 input using Image J. (NIH) software.

### Gene expression analysis

Total RNA was extracted from non-target control and MYPT1 silenced HepG2 cells using TRIzol reagent (Invitrogen) as described in the manufacturer’s protocol. NanoDrop ND-1000 spectrophotometer (Thermo Scientific) was used to determine the RNA concentration. RNA was treated with RNase free DNase I (Thermo Scientific) and reverse transcription was performed with qPCRBIO cDNA synthesis kit (PCR Biosystems). PCR amplification was carried out with Maxima Hot Start PCR Master Mix (Thermo Scientific) based on manufacturer’s protocol. RT-PCR products were analyzed by agarose gel electrophoresis. The expression level of the genes of interest were performed by Image J. 1.46, normalized to GAPDH and represented as relative numbers. The primer pairs used in the RT-PCR experiments are summarized in Table S8.

### Microarray processing and data analysis

Expression pattern of non-target and MYPT1 silenced HepG2 cells was examined and analysed as detailed before^53^ using total RNA extracted as described in the previous section. Statistically significant difference was considered at p < 0.05 and fold change cut off value was 1.5.

### LC-MS/MS analysis

Bands of interest were subjected to in-gel digestion using side-chain protected porcine trypsin (37 °C, 4 h) [ http://msf.ucsf.edu/ingel.html]. For the identification of MYPT1 interacting proteins, the resulting peptide mixtures were analyzed directly by data-dependent „triple play” LC-MS/MS using a 3D-ion trap mass spectrometer (LCQ-Fleet, Thermo). Identification of MYPT1 interacting proteins and the PRMT5 phosphorylation sites from LC-MS/MS data were detailed in the Supplementary Methods section.

### Tissue array analysis

SomaPlex reverse phase protein microarray slides containing human liver and normal tissue samples from 25 clinical cases and 15 common cancer cell line lysates in triplicates were purchased from Protein Biotechnologies. Microarray slides were handled following the manufacturer’s instructions as detailed in Supplementary Methods. Dots of interest were analysed by densitometry and were all normalized to their adequate tubulin internal control. Values of the phosphorylation of PRMT5 at Thr80, -MYPT at Thr850 and H2AR3me2s were normalized to PRMT5, MYPT and H2A, respectively. Values of cancer tissues were related to normal tissue data and plotted as relative numbers.

### Statistical analysis

Normalized data were analyzed using either Student’s t-test (for two groups) or analysis of variance (one-way ANOVA, for >2 groups). Post hoc testing for one-way ANOVA was determined by Tukey’s test. Parametric statistical tests were used if the assumptions of such tests were met. All normalized variables used in statistical analyses were found to be normally distributed. Tests were conducted using GraphPad for Windows. All data presented in this work represent mean ± SEM, *n* means the number of independently performed experiments.

## Additional Information

**How to cite this article**: Sipos, A. *et al*. Myosin phosphatase and RhoA-activated kinase modulate arginine methylation by the regulation of protein arginine methyltransferase 5 in hepatocellular carcinoma cells. *Sci. Rep.* **7**, 40590; doi: 10.1038/srep40590 (2017).

**Publisher's note:** Springer Nature remains neutral with regard to jurisdictional claims in published maps and institutional affiliations.

## Supplementary Material

Supplementary Material

## Figures and Tables

**Table 1:** FT-MYPT1 binding proteins of HepG2 nuclear fraction.

NCBI gi#	Protein	MW, Da	Peptide number	Function
4505317	Protein phosphatase 1 regulatory subunit 12 A isoform a (MYPT1)	115610	20	Signal transduction
13699824	Kinesin family member 11	120111	7	Motor protein, mitosis
338443	Beta-spectrin	275259	3	Protein targeting
20070220	Protein arginine methyltransferase 5 isoform a	73322	8	Transcription regulation
4507361	Mitogen-activated protein kinase kinase kinase 7 isoform A	64930	3	Signal transduction, transcription regulation
4506583	Replication protein A1	68723	2	DNA replication, DNA damage
4505995	Protein phosphatase 1B isoform 1	53180	6	Signaling pathways, cell stress response pathways
307383	RNA helicase A	143405	3	Translation initiation, splicing, spliceosome assembly
29881667	Splicing factor proline/glutamine-rich	76255	8	Splicing
11067747	CDC5-like protein	92422	2	Cell cycle control, transcription regulation
288100	Initation factor 4B	69240	2	Translation initiation
4885225	Ewing sarcoma breakpoint region 1 isoform 2	68721	2	Gene expression, cell signaling, RNA processing
693937	Polyadenylate binding protein II	58709	12	Gene expression, RNA processing
56237027	Insulin-like growth factor 2 mRNA binding protein 1 isoform 1	63783	2	Translation regulation, mRNA transport
4504447	Heterogeneous nuclear ribonucleoprotein A2/B1 isoform A2	36041	3	Gene expression, mRNA processing
4504445	Heterogeneous nuclear ribonucleoprotein A1 isoform a	34289	2	mRNS processing and transport, RNA splicing
356168	Histone H1b	21721	2	DNA-binding, nucleosome assembly
