# Supplementary material for: Myosin phosphatase and RhoA-activated kinase modulate arginine methylation by the regulation of protein arginine methyltransferase 5 in hepatocellular carcinoma cells
Source: Sci Rep. 2017 Jan 11;7:40590. doi: 10.1038/srep40590 (PMC5225440; doi:10.1038/srep40590)
Supplement: Supplementary Material [file srep40590-s1.doc]

**SUPPLEMENTARY DATA**

**Myosin phosphatase and RhoA-activated kinase modulate arginine methylation by the regulation of protein arginine methyltransferase 5 in hepatocellular carcinoma cells**

Adrienn Sipos^1^, Judit Iván^1,2^, Bálint Bécsi^1,2^, Zsuzsanna Darula^3^, István Tamás^1^, Dániel Horváth^1^, Katalin F Medzihradszky^3^, Ferenc Erdődi^1,2^, Beáta Lontay^1*^

^1^Department of Medical Chemistry and **^¶^**^2^MTA-DE Cell Biology and Signaling Research Group, Faculty of Medicine, University of Debrecen, Debrecen, H-4032 Hungary; ^3^Laboratory of Proteomics Research, Biological Research Centre, Hungarian Academy of Sciences, H-6726, Szeged, Hungary

**Supplementary results**

***Myosin phosphatase is localized to the chromatin and the spliceosomes in the nucleus of HepG2 cells***

Initial reports have already described the presence of MYPT1 in the nucleus but its exact subnuclear localization and function remained unknown. We determined the subcellular localization of MYPT1 along with PP1cα, PP1cγ1, PP1cβ/δ isoforms in human hepatocarcinoma cells (HepG2) using antibodies specific for MYPT1, for PP1cδ or for both PP1cα and PP1cγ1[^3^](#_ENREF_3). MYPT1, PP1cα/γ1 and PP1cδ  were distributed in the cytoplasmic and nuclear subcellular fractions (Fig. 1SA). The purity of cytosol and nuclear extracts was verified by subcellular fraction-specific antibodies such as α-tubulin and lamin A/C. PP1 and PP2A enzyme activities of HepG2 lysate as well as the cytosolic and nuclear fractions were determined using ^32^P-labeled myosin light chain (^32^P-MLC20) which was shown to be a substrate of both enzymes[^4^](#_ENREF_4) (Fig. 1SB). Protein phosphatase activity of the cytosolic fraction was more than 20% higher than that of the nuclear fraction. To differentiate between PP1 and PP2A activity the phosphatase activity of fractions was measured in the presence of 2 μM inhibitor-2 (I2), which is a highly selective inhibitor of PP1, but does not influence PP2A activity[^5^](#_ENREF_5). Specific activity measured in the presence of 2 μM I2 suggested that ~60% of total phosphatase activity of nuclear fraction was related to PP1, while PP2A was in excess in the cytosolic fraction and represented ~75% of the phosphatase activity. We also examined the subcellular localization of PP1δ isoforms and MYPT1 in HepG2 cells by fluorescent confocal microscopy (Fig. 1SC). In agreement with the results of immunoblotting, PP1cδ isoform was detected in the cytoplasm and in the nucleus as well. Nuclear colocalization of MYPT1 with PP1cδ  (Fig. 1SC, bottom panel) was observed indicating the nuclear presence of MP. The study of subnuclear localization of MYPT1 using the anti-MYPT1^1-296^ antibody and subnuclear compartment-specific antibodies showed that MYPT1 accumulated in spliceosomes and colocalized with histone H1b protein (Fig. 1SC, middle panels) but did not localize to the nuclear membrane or nucleoli (Fig. 1SC, upper panels). These data suggest that MYPT1 is a putative PP1-targeting subunit during pre-mRNA splicing and that it may plays a role in the regulation of chromatin structure.

**Supplementary materials and methods**

*Chemicals and antibodies*

All chemicals were obtained from Sigma Aldrich unless indicated otherwise. Antibodies were as follows: anti-MYPT^1-296^[^3^](#_ENREF_3); -PP1cδ, -nucleolemma, -nucleolus, -spliceosome, -PRMT5, pMYPT^T850^  from Upstate; anti-MYPT1 (E-19), -lamin A/C, monoclonal anti-α-tubulin from Santa Cruz Biotechnology; anti-H2AR3me2s from Abcam; anti-MEP50, -phospho-Thr, -histone H2A, -histone H4, -H4R3me2s and HRP-conjugated anti-mouse IgG from Cell Signaling Technology; anti-c-Myc and horseradish-peroxidase (HRP) conjugated anti-rabbit IgG from Sigma Aldrich; anti-retinoblastoma protein from BD Biosciences, HRP-conjugated Clean-blot IP detection reagent from Thermo Scientific Inc.; Alexa Fluor 633-conjugated Phalloidin, Alexa Fluor 488-conjugated anti-rabbit IgG , Alexa Fluor 546-conjugated anti-goat and -mouse IgG from Molecular Probes. Rabbit polyclonal affinity purified phospho-specific PRMT5 antibody was made by Abmart Inc. using the epitope of GRDWN(pT)LIVGK and validated by dot blot following manufacturer’s suggestions (ab-mart.com). The phospho-Thr80 specific antibody recognized neither the wt nor the T80A mutant peptide of PRMT5 only the Thr80 phosphorylated PRMT5 synthetic peptide by dot blot analysis (Fig. S2F). The antibody was also found specific to the ROK-phosphorylated Flag(FT)-PRMT5 but had no cross-reaction with either the non-phosphorylated FT-PRMT5 or the non-phosphorylated and phosphorylated form of FT- PRMT5-Thr80Ala mutant (Fig. S2G). The phospho-PRMT5^Thr80^ antibody was further tested by treating HepG2 cells with (W/PI) or without (WO/PI) phosphatase inhibitors (Fig. S2H). The data suggest that the full inhibition of protein phosphatases with phosphatase inhibitors dramatically increased the Thr80 phosphorylation of PRMT5 while without PI the phosphorylation was completely dissolved. None of the treatment had any significant effect on the protein expression level of PRMT5.

*Transient transfections*

Transfection mixture containing 100 nM siRNAs and Dharmafect 2 reagent (Thermo Scientific Inc.) was added to serum-free medium comprising HepG2 cells. After 30 min, the medium was completed with 10% FBS and cells were incubated for 6 h and transfection medium was replaced to complete medium. After 42 h of incubation cells were applied. TsA201 cells were transfected with pReceiver-M11 plasmids containing FT-PRMT5wt, FT-PRMT5^T80A^ and FT-MYPT1 inserts using 1 mg/ml polyethylenimine (PEI) transfection reagent (Polysciences Inc.) in serum-free medium. After 6 h incubation medium was replaced to complete DMEM and after an additional 24 h cells were lysed.

*Membrane stripping for reprobing*

Membranes were washed four times for 5 minutes each in TBS completed with Tween (TBST) and were incubated from 30 minutes at 50^o^C in stripping buffer with slight agitation. Then membranes were washed six times for 5 minutes with TBST.

*Immunoprecipitation and pull-down assays*

FT-PRMT5wt, -PRMT5^T80A^ and FT-MYPT1 was purified from transfected tsA201 lysates using anti-Flag M2 affinity gel (Sigma Aldrich) applying the manufacturer’s protocol. Flag-PRMT5wt and -PRMT5^T80A^ proteins were bound to anti-FT M2 affinity gel during kinase and phosphatase assays and *in vitro* protein arginine methyltransferase assays. FT-MYPT1 was eluted from the beads with 300 μg/ml Flag-peptide from Sigma Aldrich for use in phosphatase and *in vitro* protein arginine methyltransferase assays.

*Identification of MYPT1 interacting proteins from LC-MS/MS data*

Peak lists generated from the MS/MS data by the Mascot Distiller software (v2.2.1.0) were searched against the human subset of the NCBI database (downloaded 02/20/2010; 183553 protein sequences) using the Mascot search engine. Search parameters: enzyme: trypsin with maximum 2 missed cleavage sites per peptide; fixed modification: carbamidomethyl (Cys); variable modifications: acetylation (protein N-terminus), oxidation (Met), pyroglutamic acid formation (peptide N-terminal Gln); mass accuracy: 0.6 Da and 1 Da for precursor and fragment ions (both monoisotopic), respectively. Acceptance parameters: peptide score>38 (p<0.05), minimum 2 unique peptides/protein (See complete list in Supplementary Table 1).

*Identification of PRMT5 phosphorylation sites from LC-MS/MS data*

Peak lists generated from the MS/MS data by the PAVA software (v2010/september) were searched against the Swissprot database (downloaded 06/27/2013; 540546 protein sequences) using the ProteinProspector search engine. Search parameters: enzyme: trypsin with maximum 1 missed cleavage site per peptide; fixed modification: carbamidomethyl (Cys); variable modifications: acetylation (protein N-terminus), oxidation (Met), pyroglutamic acid formation (peptide N-terminal Gln) allowing maximum 2 variable modifications per peptide; mass accuracy: 5 ppm and 0.6 Da for precursor and fragment ions (both monoisotopic), respectively. Subsequently another search was conducted on the subset of confidently identified proteins using the same search parameters except that maximum 2 missed cleavages per peptides were allowed, and phosphorylation on Ser/Thr/Tyr was also set as variable modification allowing maximum 3 variable modifications/peptides. For all searches the following acceptance criteria were applied: score>22 and 15, and E-value<0.01 and 0.05 for protein and peptide identifications, respectively. For phosphopeptide site assignments, SLIP threshold [^6^](#_ENREF_6) was set to 6.

*Phosphatase activity assay*

Protein phosphatase activity of total lysate, cytosolic and nuclear extracts of HepG2 cells (at 0.05 mg/ml final concentration) were determined using 1 μM ^32^P-labelled 20 kDa light chain of myosin (^32^P-MLC20) as a substrate as detailed previously [^7^](#_ENREF_7). Briefly, the reaction was initiated by addition of the substrate and after 1.5 min incubation was terminated by the addition of 200 μl 10 % TCA and 200 μl 6 mg/ml BSA. After centrifugation the ^32^P_i_ released from the substrate in the supernatant was determined in a scintillation counter.

*Tissue array analysis*

SomaPlex reverse phase protein microarray slides were blocked by 3% bovine serum albumin dissolved in TBST assayed with anti-phospho-PRMT5^T80^, -phospho-MYPT^T850^ and -histone H2A antibodies at 4°C. After washing slides were incubated with HRP-labelled secondary antibodies for 2 hours and the antibody binding was developed by enhanced chemiluminescence (ECL) using x-ray films. Each slide was stripped and incubated by antibodies raised against PRMT5, MYPT^1-296^, H2AR3me2s and α-tubulin.

*Buffers*

PBS: pH7.2; 2.7 mmol/L KCl, 1.5 mmol/L KH_2_PO_4_, 136.9 mmol/L NaCl, 8.9 mmol/L Na_2_HPO_4_·7H_2_O

lysis buffer for tsA cells: 50 mM Tris HCl, pH 7.4, 150 mM NaCl, 1 mM EDTA, 1% (v/v) Triton X-100

lysis buffer for MTT assay: 10 mM HEPES, 0.1% CHAPS, 5 mM dithiothreitol, 2 mM EDTA, 1 x concentrated protease inhibitor cocktail and 1 mM PMSF, pH 7.4

buffer A: 10 mM Hepes, pH 7.9, 10 mM KCl, 0.1 mM EDTA, 0.1 mM EGTA, 1 mM DTT, 0.5 % (v/v) Nonidet P-40, 0.5 mM PMSF, 1 x concentrated protease inhibitor cocktail

buffer B: 20 mM Hepes, pH 7.9, 420 mM NaCl, 0.5 mM EDTA, 0.5 mM EGTA, 1 mM DTT, 0.5 mM PMSF, 1 x concentrated protease inhibitor cocktail

TBS: 50 mM Tris-HCl, pH 7.4, 150 mM NaCl

stripping buffer: 62 mM Tris, 2% (m/v) SDS, 0.7% (v/v) 2-mercaptoethanol, pH 6.8

buffer C: 20 mM MOPS, pH 7.2, 25 mM beta-glycerophosphate, 0.5 mM EGTA, 0.5 mM DTT, 5 mM MgCl_2_, 1 μM mycrocystin-LR (MC-LR)

PKA assay buffer: 50 mM HEPES, pH 6.4, 6 mM MgCl_2_, 1 mM EGTA, 10 mM NaF, 1 μM MC-LR

PKC assay buffer20 mM HEPES, 10 mM MgCl_2_, 1 mM DTT, 0.03% Triton-X100 and 0.65 mM CaCl_2_ : pH 7.5 plus micelles

caspase reaction buffer: 100 mM HEPES, 10% sucrose, 5 mM dithiothreitol, 0.1% CHAPS, pH 7.25

IP buffer: 30 mM Tris-HCl, pH 7.0, 150 mM NaCl, 1 mM EDTA, 0.1% Triton X-100 with 1 x concentrated protease inhibitor cocktail

50x phosphatase inhibitor cocktail: 50 mM NaF, 50 mM Na_4_P_2_O_7_, 50 mM β-glicerophosphate, 50 mM Na_3_VO_4_, 1 mM EDTA, 1 μM MC-LR

**Supplementary figure legends**

**Figure S1. Subcellular distribution of MYPT1 in HepG2 cells.** (A) HepG2 whole cell lysate (Wl), cytosol (Cs) and nuclear (Nuc) fractions were analysed by Western blot using antibodies specific for MYPT1, PP1cδ and PP1cα/γ as well as for subcellular fraction-specific markers such as α-tubulin for cytosol and Lamin A/C for nucleus. Samples derived from the same experiment and were processed in parallel. (B) Protein phosphatase activity of cytosolic and nuclear fractions was determined in the absence and in the presence of 2 μM inhibitor-2 using ^32^P-MLC20 as substrate. Values represent mean ± SEM; n=3; *p<0.05, **p<0.01, ***p<0.001 by student *t-*test. (C) Subnuclear distribution of MYPT1 by immunofluorescent microscopy using anti-MYPT1^1-296^ (green) or anti-MYPT1 (E19) (red), anti-histone H1 (green), anti-PP1cδ (green) and subnuclear-specific markers such as anti-nucleolemma (red), anti-nucleoli (red), anti-spliceosome (red) specific antibodies. Nucleus is stained with DAPI (blue). Scale bars: 20 μm.

**Figure S2. Purity of recombinant proteins and validation of anti-pPRMT5^T80^ antibody.** Coomassie stained gels represent purity of GST-MYPT1^1-1004^, GST_MYPT1^667-1004^ (A), FT--PRMT5 (Sino Biological) and immunoprecipitated (IP’d) FT PRMT5 (B) MEP50-free FT-His-PRMT5 and Flag IP’d PRMT5 in complex with MEP50 (C), rPP1cδ (D) and IP’d FT-MYPT1 (E). Validation of anti-PRMT5 and anti-phospho-PRMT5^T80^ antibodies by dot blot analysis using wild type (T80wt), alanine mutant (T80A) and phosphorylated wild type (pT80) synthetic peptides of PRMT5 from Abmart (F). BSA and anti-rabbit IgG was applied as negative and positive control, respectively. Validation of anti-phospho-PRMT5^T80^ antibody by immunoblot using non-phosphorylated and ROK-phosphorylated FT-PRMT5wt and FT-PRMT5^T80A^ proteins (G). The amount of PRMT5 was detected by PRMT5 specific antibody. Immunoblot of HepG2 total lysate prepared in the absence (wo/PI) or in the presence of phosphatase inhibitors (w/PI) analysed by anti-phospho-PRMT5^T80^ (left panel) and anti-PRMT5 antibody (right panel) (H).

**Figure S3. Phosphorylation of wild type and alanine mutant of PRMT5^T80^.** Wild type (A) and alanine mutant (B) of PRMT5^T80^ (PRMT5^T80A^) was analysed by western blot using anti-phospho-PRMT5^T80^ and PRMT5 antibody after *in vitro* phosphorylation by ROK in the presence or in the absence of 10 μM H1152, a selective ROK inhibitor.

**Figure S4. Effect of MYPT1 silencing on cell survival of HepG2 cells**. Viability (A) and Caspase-3 activity (B) of untreated (Ctrl), non-target control (NT ctrl) and MYPT1-silenced (siMYPT1) HepG2 cells were determined by MTT or Caspase-3 activity assays. Values obtained for untreated control cells were taken as 100%. Values represent mean ± SEM; n=3.

**Figure S5. PRMT5 activity of HepG2 and MCF7 nuclear extracts.** Quantitative PRMT5 activity of nuclear fractions from non-target control (Ctrl) and MYPT1-silenced (siMYPT1) HepG2 (A) and MCF7 (B) cells was measured by colorimetric Epigenase PRMT Methyltransferase (Type II-Specific) assay kit. Activity is expressed in optical density (OD)/min/mg.

**Figure S6. Extracted ion chromatograms of PRMT5 phosphopeptides.** PRMT5 phosphorylation sites were determined in control and ROK-phosphorylated samples. Extracted ion chromatograms of upper panel: m/z 656.3381 representing the triply charged SDLLLSGRDWNT(Phospho)LIVGK peptide, phospho-[69-85] middle panel:m/z 616.3143 representing the triply charged NRPGPQTRSDLLLSGR peptide phosphorylated on either Thr-67 or Ser-69, phospho-[69-85]lower panel: m/z 784.7137 representing the triply charged VSSGRDLNCVPEIADTLGAVAK peptide phosphorylated on either Ser-15 or Ser-16, phospho-[14-35]of PRMT5 in the control and ROK treated PRMT5 samples. NL values on the right indicate the maximum of the absolute intensity of the m/z values investigated.

**Supplementary tables**

**Table S1. FT-MYPT1 binding proteins of HepG2 nuclear fraction**

| **NCBI gi#** | **Protein** | **Peptides** |
| --- | --- | --- |
| 4505317 | Protein phosphatase 1 regulatory subunit 12A isoform a (MYPT1) | 63 GADINYANVDGLTALHQACIDDNVDMVK 90  162 QGVDIEAAR 170  162 QGVDIEAARKEEER 175  183 QWLNSGHINDVR 194  250 ILVDNLCDMEMVNK 263  299 SPLIESTANMDNNQSQK 315  319 NKETLIIEPEKNASR 333  442 KTGSYGALAEITASK 456  443 TGSYGALAEITASK 456  494 LAYVAPTIPR 503  505 LASTSDIEEKENR 517  558 RQDDLISSSVPSTTSTPTVTSAAGLQK 584  585 SLLSSTSTTTK 595  694 RSTQGVTLTDLQEAEKTIGR 713  695 STQGVTLTDLQEAEK 709  695 STQGVTLTDLQEAEKTIGR 713  908 SGSYSYLEER 917  908 SGSYSYLEERKPYSSR 923  935 LYEQILAENEK 945 |
| 13699824 | Kinesin family member 11 | 147 LTDNGTEFSVK 157  222 TTAATLMNAYSSR 234  258 IGKLNLVDLAGSENIGR 274  261 LNLVDLAGSENIGR 274  298 VITALVER 305  319 ILQDSLGGR 327  811 ISQETEQRCESLNTR 825 |
| 338443 | Beta-spectrin | 444 LVSQDNFGFDLPAVEAATK 462  905 VAVVNQIAR 913  1548 SQNIVTDSSSLSAEAIR 1564 |
| 20070220 | Protein arginine methyltransferase 5 isoform a | 2 AAMAVGGAGGSR 13  19 DLNCVPEIADTLGAVAK 35  69 SDLLLSGRDWNTLIVGK 85  202 IAVALEIGADLPSNHVIDR 220  228 AAILPTSIFLTNK 240  334 YSQYQQAIYK 343  369 GPLVNASLR 377  386 IKLYAVEK 393 |
| 4507361 | Mitogen-activated protein kinase kinase kinase 7 isoform A | 72 KAFIVELR 79  412 SIQDLTVTGTEPGQVSSR 429  520 KQELVAELDQDEKDQQNTSR 539 |
| 4506583 | Replication protein A1 | 184 VVPIASLTPYQSK 196  490 VIDQQNGLYR 499 |
| 4505995 | Protein phosphatase 1B isoform 1 | 91 SGSALELSVENVK 103  91 SGSALELSVENVKNGIR 107  130 SGSTAVGVMISPK 142  180 IQNAGGSVMIQR 191  201 ALGDYDYK 208  361 NVIEAVYSR 369 |
| 307383 | RNA helicase A | 64 DFVNYLVR 71  435 AAECNIVVTQPR 446  837 ELDALDANDELTPLGR 852 |
| 29881667 | Splicing factor proline/glutamine-rich | 299 LFVGNLPADITEDEFK 314  320 YGEPGEVFINK 330  320 YGEPGEVFINKGK 332  343 ALAEIAKAELDDTPMR 358  366 FATHAAALSVR 376  414 GIVEFASKPAAR 425  480 FAQHGTFEYEYSQR 493  667 FGQGGAGPVGGQGPR 681 |
| 11067747 | CDC5-like | 207 GVDYNAEIPFEK 218  478 LGLLGLPAPK 487 |
| 288100 | Initation factor 4B | 166 IRVDVADQAQDKDR 179  357 AASIFGGAKPVDTAAR 372 |
| 4885225 | Ewing sarcoma breakpoint region 1 isoform 2 | 269 QDHPSSMGVYGQESGGFSGPGENR 292  411 GDATVSYEDPPTAK 424 |
| 693937 | Polyadenylate binding protein II | 25 RSLGYAYVNFQQPADAER 42  26 SLGYAYVNFQQPADAER 42  43 ALDTMNFDVIK 53  70 KSGVGNIFIK 79  71 SGVGNIFIK 79  114 GYGFVHFETQEAAER 128  162 AKEFTNVYIK 171  207 GFGFVSFER 215  266 YQGVNLYVK 274  287 KEFSPFGTITSAK 299  288 EFSPFGTITSAK 299  491 SKVDEAVAVLQAHQAK 506 |
| 56237027 | Insulin-like growth factor 2 mRNA binding protein 1 isoform 1 | 200 LLVPTQYVGAIIGK 213  526 DQTPDENDQVIVK 538 |
| 4504447 | Heterogeneous nuclear ribonucleoprotein A2/B1 isoform A2 | 192 GGNFGFGDSR 201  202 GGGGNFGPGPGSNFR 216  314 NMGGPYGGGNYGPGGSGGSGGYGGR 338 |
| 4504445 | Heterogeneous nuclear ribonucleoprotein A1 isoform a | 16 LFIGGLSFETTDESLR 31  285 SSGPYGGGGQYFAKPR 300 |
| 356168 | Histone H1b | 54 SGVSLAALKK 63  64 ALAAAGYDVEK 74 |

**Table S2. Percentage distribution of FT-MYPT1 binding proteins by their cellular roles**

| **Cellular role** | **Percentage (%)** |
| --- | --- |
| RNA processing, splicing, spliceosome assembly | 27.58 |
| Gene expression | 17.24 |
| Transcription regulation | 10.34 |
| Translation regulation | 10.34 |
| Signal transduction | 13.79 |
| DNA-binding, nucleosome assembly | 3.44 |
| DNA replication, DNA damage | 3.44 |
| Others (mitosis, protein targeting, protein refolding, embryogenesis, cell proliferation, cell cycle control) | 13.79 |

**Table S3. Primer pairs of PRMT5 site-directed mutagenesis**

| **PRMT5 mutant** | **Forward primer (5’ to 3’)** | **Reverse primer (5’ to 3’)** |
| --- | --- | --- |
| PRMT5^T80A^ | TGTCAGGAAGGGACTGGAATGCGCTAATTGTGGG | CCCACAATTAGCGCATTCCAGTCCCTTCCTGACA |

**Table S4**. Primers for DNA sequencing of PRMT5 mutants

| PRMT5 forward # 1 353 | 5’ – GTCTTCCAGCTTTCCTGCTG – 3’ |
| --- | --- |
| PRMT5 forward # 2 858 | 5’ – CTTAAGCCAGAACCGTCCTC – 3’ |
| PRMT5 forward # 3 1357 | 5’ – GCCCAGCACTTCCTAAAAGA – 3’ |
| PRMT5 reverse 447 | 5’ – GGAAGAGTGATGGCCAGTGT – 3’ |

**Table S5. siRNA sequences for MYPT1 silencing**

siRNA #1: 5’ – CAACUAAACAGGCCAAAUA – 3’

siRNA #2: 5’ – GCUAAAUAGUGGUCAUAUA – 3’

siRNA #3: 5’ – ACAAAGAGACGUUGAUUAU – 3’

siRNA #4: 5’ – CGGAUUCCAUUUCUAGAUA – 3’

**Table S6. Mean intensity of immunofluorescent staining**

| **Detected protein** | **Mean intensity in control cells** | **Mean intensity in siMYPT cells** |
| --- | --- | --- |
| symmetrical dimethylated H2Ar3 | 12.8 | 21.79 |
| H2A | 18.2 | 20.7 |
| symmetrical dimethylated H4r3 | 8.19 | 22.58 |
| H4 | 15.7 | 13.4 |
|  | **Colocalization rate in control cells** | **Colocalization rate in siMYPT cells** |
| PRMT5 Thr80 to DAPI | 8.79 | 28.17 |
| PRMT5 Thr80 to F actin | 56.78 | 41.26 |

**Table S7. List of differentially expressed genes in siMYPT1 HepG2 with 1.5 fold change cut-off limit.**

| **Gene symbol** | **Gene description** | **Regulation** | **Fold change** |
| --- | --- | --- | --- |
| PPP1R12A | protein phosphatase 1, regulatory (inhibitor) subunit 12A | down | 2.0050275 |
| EPHA2 | EPH receptor A2 | up | 1.5645913 |
| SLC2A1 | solute carrier family 2 (facilitated glucose transporter), member 1 | up | 1.6917205 |
| TGFBR3 | transforming growth factor, beta receptor III | up | 1.5754458 |
| TMCO1 | transmembrane and coiled-coil domains 1 | down | 1.9577281 |
| KLF6 | Kruppel-like factor 6 | up | 1.6079206 |
| ANKRD1 | ankyrin repeat domain 1 (cardiac muscle) | up | 2.1838295 |
| HEPACAM|HEPN1 | hepatocyte cell adhesion molecule | HEPACAM opposite strand 1 | down | 1.6288424 |
| OAS1 | 2',5'-oligoadenylate synthetase 1, 40/46kDa | up | 1.6448771 |
| SLC2A1 | solute carrier family 2 (facilitated glucose transporter), member 1 | up | 1.6917205 |
| TGFBR3 | transforming growth factor, beta receptor III | up | 1.5754458 |
| TMCO1 | transmembrane and coiled-coil domains 1 | down | 1.9577281 |
| KLF6 | Kruppel-like factor 6 | up | 1.6079206 |
| ANKRD1 | ankyrin repeat domain 1 (cardiac muscle) | up | 2.1838295 |
| HEPACAM|HEPN1 | hepatocyte cell adhesion molecule | HEPACAM opposite strand 1 | down | 1.6288424 |
| OAS1 | 2',5'-oligoadenylate synthetase 1, 40/46kDa | up | 1.6448771 |
| DUSP6 | dual specificity phosphatase 6 | up | 1.5159508 |
| LPAR6 | lysophosphatidic acid receptor 6 | up | 1.5162095 |
| IRF9 | interferon regulatory factor 9 | up | 1.6238389 |
| IFI27 | interferon, alpha-inducible protein 27 | up | 1.6448994 |
| KIAA1199 |  | up | 2.1050441 |
| SNORD68|RPL13 | small nucleolar RNA, C/D box 68 | ribosomal protein L13 | up | 1.7279054 |
| MIR21 | microRNA 21 | up | 1.9698615 |
| KRT23 | keratin 23 (histone deacetylase inducible) | up | 1.5333802 |
| SLC16A6 | solute carrier family 16, member 6 (monocarboxylic acid transporter 7) | up | 2.1183825 |
| DTNA | dystrobrevin, alpha | up | 1.6605202 |
| ICAM1 | intercellular adhesion molecule 1 | up | 1.5522647 |
| GDF15 | growth differentiation factor 15 | up | 1.6862459 |
| MFSD2B | major facilitator superfamily domain containing 2B | down | 1.519762 |
| SNX17 | sorting nexin 17 | down | 1.6879824 |
| FETUB | fetuin B | down | 1.7819421 |
| KNG1 | kininogen 1 | down | 1.505488 |
| FILIP1L | filamin A interacting protein 1-like | up | 1.5426576 |
| SLC7A11 | solute carrier family 7, (cationic amino acid transporter, y+ system) member 11 | up | 1.6259567 |
| ITGA2 | integrin, alpha 2 (CD49B, alpha 2 subunit of VLA-2 receptor) | up | 1.794632 |
| EGR1 | early growth response 1 | up | 1.6131204 |
| SPRY4 | sprouty homolog 4 (Drosophila) | up | 1.6954104 |
| GMDS | GDP-mannose 4,6-dehydratase | up | 1.5297856 |
| IER3 | immediate early response 3 | up | 2.0160716 |
| SERPINE1 | serpin peptidase inhibitor, clade E (nexin, plasminogen activator inhibitor type 1), member 1 | up | 1.6623654 |
| C8orf4 | chromosome 8 open reading frame 4 | up | 1.5722965 |

**Table S8.** RT-PCR primer pairs

| **Target** | **Forward primer (5’ to 3’)** | **Reverse primer (5’ to 3’)** |
| --- | --- | --- |
| PPP1R12A | CCACAACCCTGACTACAACTAC | TCTCCTTCTTTCTCCTCTTCTCT |
| PRMT5 | CGGAGAAGGGCAGACTA | CAATTTCAAGAGCCACTGC |
| RAP1A | ATAAGAGTATGTGTCTCACTGC | TCTTCATTCCTGTAATCTGGC |

**Supplementary figures**

**Figure S1.**


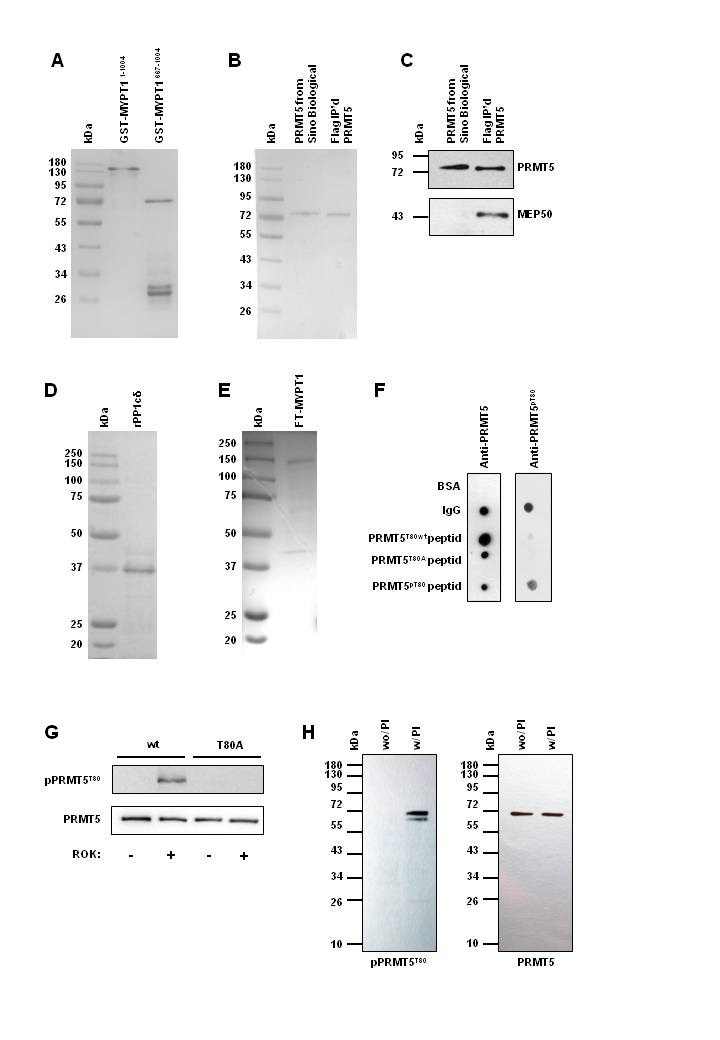
**Figure S2.**


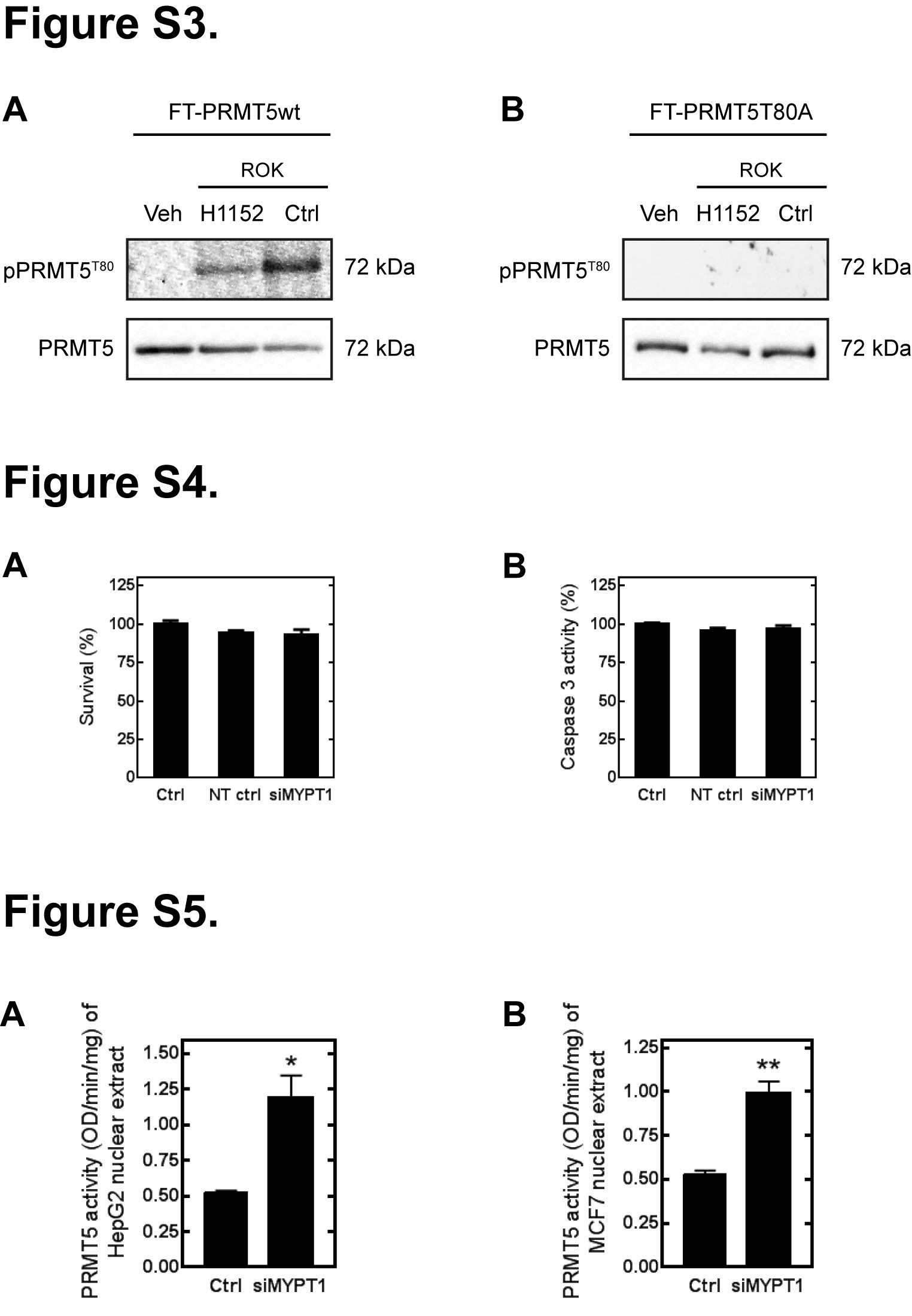
**Figure S3.**


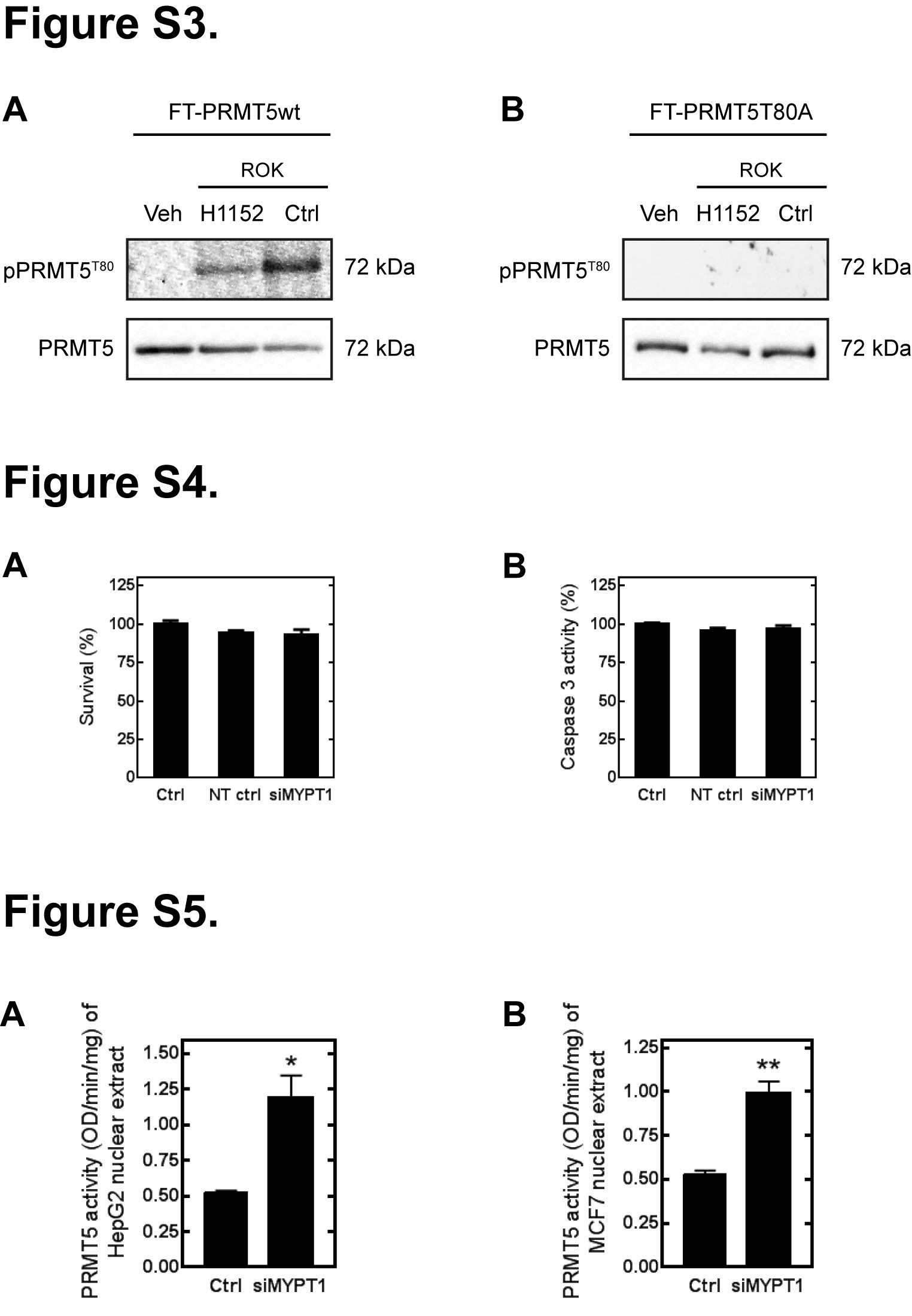
**Figure S4.**


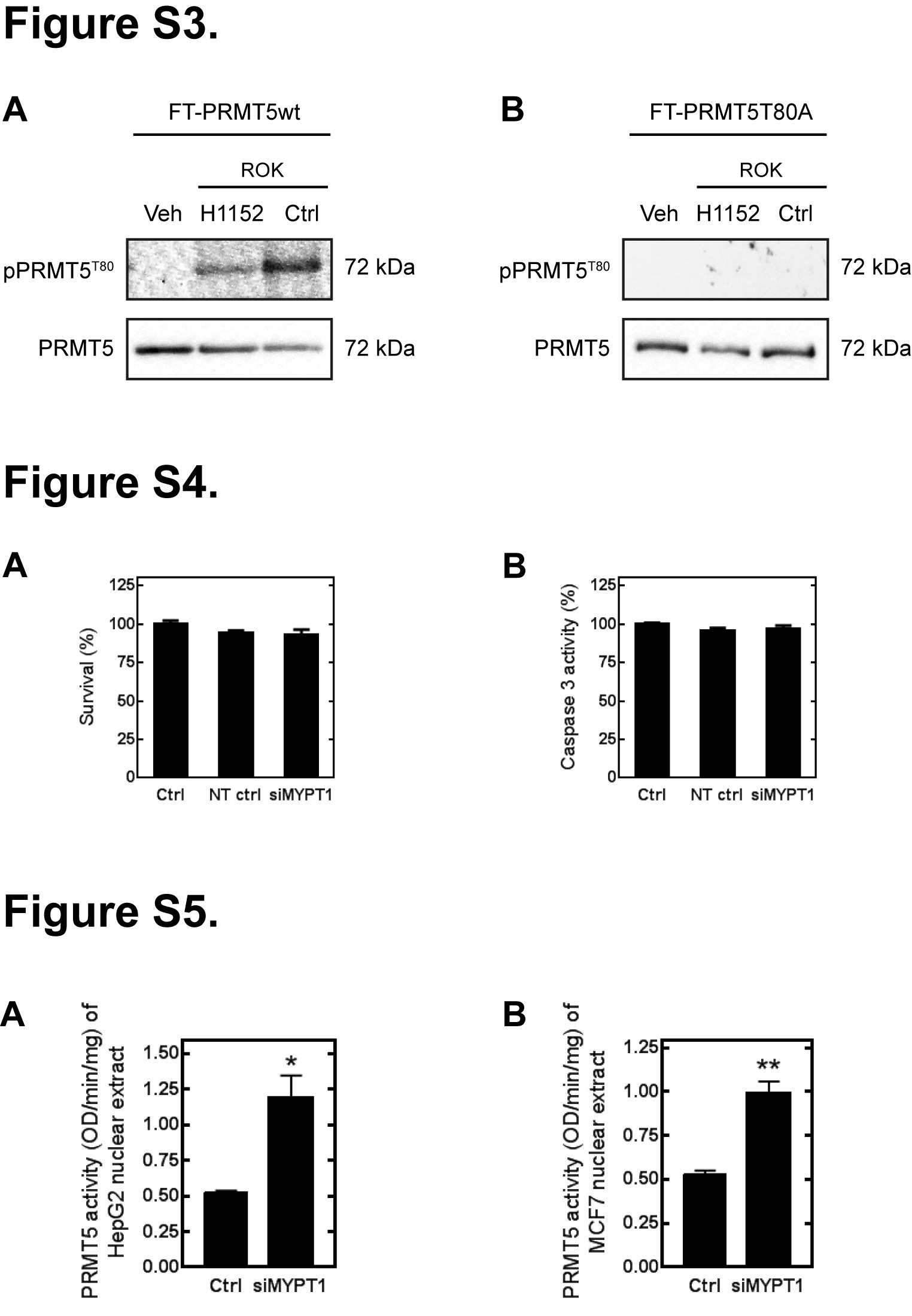
**Figure S5.**

**Figure S6.**

**
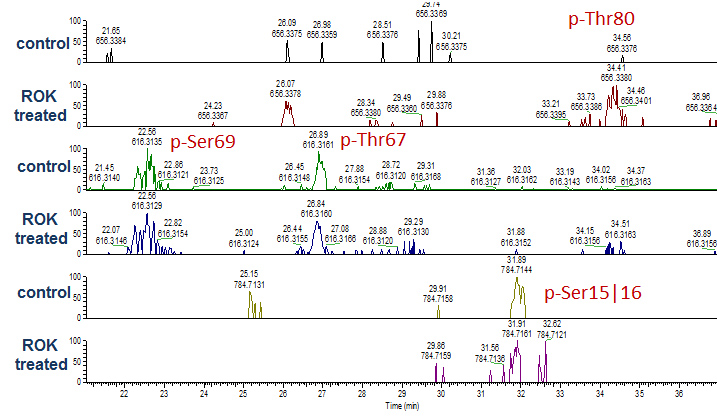
**

**Supplementary references**

1 Lontay, B., Kiss, A., Gergely, P., Hartshorne, D. J. & Erdodi, F. Okadaic acid induces phosphorylation and translocation of myosin phosphatase target subunit 1 influencing myosin phosphorylation, stress fiber assembly and cell migration in HepG2 cells. *Cell Signal* **17**, 1265-1275, doi:10.1016/j.cellsig.2005.01.008 (2005).

2 Tran, H. T., Ulke, A., Morrice, N., Johannes, C. J. & Moorhead, G. B. Proteomic characterization of protein phosphatase complexes of the mammalian nucleus. *Molecular & cellular proteomics : MCP* **3**, 257-265, doi:10.1074/mcp.M300115-MCP200 (2004).

3 Lontay, B. *et al.* Localization of myosin phosphatase target subunit 1 in rat brain and in primary cultures of neuronal cells. *J Comp Neurol* **478**, 72-87, doi:10.1002/cne.20273 (2004).

4 Dedinszki, D. *et al.* Protein phosphatase-1 is involved in the maintenance of normal homeostasis and in UVA irradiation-induced pathological alterations in HaCaT cells and in mouse skin. *Biochimica et biophysica acta* **1852**, 22-33, doi:10.1016/j.bbadis.2014.11.005 (2015).

5 Sheng, X. & Wang, Z. Protein arginine methyltransferase 5 regulates multiple signaling pathways to promote lung cancer cell proliferation. *BMC Cancer* **16**, 567, doi:10.1186/s12885-016-2632-3 (2016).

6 Baker, P. R., Trinidad, J. C. & Chalkley, R. J. Modification site localization scoring integrated into a search engine. *Molecular & cellular proteomics : MCP* **10**, M111 008078, doi:10.1074/mcp.M111.008078 (2011).

7 Erdodi, F. *et al.* Endothall thioanhydride inhibits protein phosphatases-1 and -2A in vivo. *The American journal of physiology* **269**, C1176-1184 (1995).
